# Modification of *BCLX* pre-mRNA splicing has antitumor efficacy alone or in combination with radiotherapy in human glioblastoma cells

**DOI:** 10.1038/s41419-024-06507-x

**Published:** 2024-02-21

**Authors:** Zhihui Dou, Huiwen Lei, Wei Su, Taotao Zhang, Xiaohua Chen, Boyi Yu, Xiaogang Zhen, Jing Si, Chao Sun, Hong Zhang, Cuixia Di

**Affiliations:** 1 Bio-Medical Research Center, Institute of Modern Physics, Chinese Academy of Sciences, Lanzhou, 730000 China; 2 Advanced Energy Science and Technology Guangdong Laboratory, Huizhou, 516029 China; 3 Key Laboratory of Heavy Ion Radiation Biology and Medicine of Chinese Academy of Sciences, Lanzhou, 730000 China; 4 College of Life Sciences, University of Chinese Academy of Sciences, Beijing, 101408 China

**Keywords:** Radiotherapy, Chemotherapy

## Abstract

Dysregulation of anti-apoptotic and pro-apoptotic protein isoforms arising from aberrant splicing is a crucial hallmark of cancers and may contribute to therapeutic resistance. Thus, targeting RNA splicing to redirect isoform expression of apoptosis-related genes could lead to promising anti-cancer phenotypes. Glioblastoma (GBM) is the most common type of malignant brain tumor in adults. In this study, through RT-PCR and Western Blot analysis, we found that *BCLX* pre-mRNA is aberrantly spliced in GBM cells with a favored splicing of anti-apoptotic Bcl-xL. Modulation of *BCLX* pre-mRNA splicing using splice-switching oligonucleotides (SSOs) efficiently elevated the pro-apoptotic isoform Bcl-xS at the expense of the anti-apoptotic Bcl-xL. Induction of Bcl-xS by SSOs activated apoptosis and autophagy in GBM cells. In addition, we found that ionizing radiation could also modulate the alternative splicing of *BCLX*. In contrast to heavy (carbon) ion irradiation, low energy X-ray radiation-induced an increased ratio of Bcl-xL/Bcl-xS. Inhibiting Bcl-xL through splicing regulation can significantly enhance the radiation sensitivity of 2D and 3D GBM cells. These results suggested that manipulation of *BCLX* pre-mRNA alternative splicing by splice-switching oligonucleotides is a novel approach to inhibit glioblastoma tumorigenesis alone or in combination with radiotherapy.

## Background

RNA splicing is a fundamental step that contribute to the high complexity of transcriptomes of multicellular eukaryotes. Dysregulation of alternative RNA splicing is a hallmark that characterizes almost all cancer types [1, 2]. Aberrant splicing in tumors results either from altered expression of key splicing regulatory proteins or from mutations in cis-regulatory elements of alternatively spliced genes [3, 4]. Cancer cells could generate tumor-specific splicing variants to promote tumorigenesis and progression. Additionally, alternatively spliced mRNA species has been found to be an important mechanism of resistance in chemotherapy, immunotherapy or radiotherapy [5–7], thus rendering cancer cells more susceptible to splicing modulation. Splicing regulation can be achieved by two promising approaches: small-molecule splicing modulators [8–10] and splice-switching oligonucleotides (SSOs) that bind specific RNA-target sequence to modulate pre-mRNA splicing [11–13], provides insight into potential splicing modulation-based cancer therapeutics on the horizon.

Apoptosis regulator Bcl-extra (*BCLX*), also named *BCL2L* or *BCL2L1*, is an essential member of *BCL2* family proteins that regulates cell fate [14]. *BCLX* nascent transcripts are alternatively spliced and produce two antagonistic isoforms. The long isoform Bcl-xL splicing at the distal end of the 5′ splice site blocks apoptosis by inhibiting pro-apoptotic counterparts of *BCL2* family, whereas the short isoform Bcl-xS splicing at the proximal end can promote apoptosis [15]. The elevated level of long isoform Bcl-xL caused by aberrant splicing has been revealed in a multitude of human cancers and considered to be a powerful driving force for apoptotic resistance [15, 16]. Bcl-xL mediated apoptotic inhibition was also the main reason that confer resistance towards chemo- and radiotherapy in tumors [17–22]. Instead, cells with highly expressed Bcl-xS was suggested to be more sensitive to apoptosis stimuli [23]. The unbalanced expression of pro-apoptotic Bcl-xS and anti-apoptotic Bcl-xL proteins plays a vital role in regulating the switch between cell life and death in many solid tumors.

Glioblastomas (GBM) is the most common malignant type of primary brain tumor in adults with the worst prognosis. The patients with a median survival of <2 years and the 5-year survival rate is less than 5% [24]. Although the standard-of care, the outlook for GBM patients remains poor and relapse within six months [25, 26], which is partly due to the high heterogeneity and the primary or acquired resistance of tumor cells to radiotherapy and chemotherapy. Thus, the clinical management of GBM patients remains a significant challenge and there is an unmet need for novel, efficacious therapies directed against defined molecular targets.

In this study, we verified that GBM cells preferentially express anti-apoptotic Bcl-xL through splicing at the proximal 5′ splice site (5’PSS) of exon 2 of *BCLX* pre-mRNA. Modulation of *BCLX* pre-mRNA splicing using splice-switching oligonucleotides to block the expression of Bcl-xL significantly decreased cell survival and increased caspase-mediated apoptosis in GBM cells. Intriguingly, we found that ionizing radiation could also modulate the alternative splicing of *BCLX*. Inhibiting Bcl-xL through splicing modulation can potently radiosensitize 2D and 3D GBM cells. Importantly, we showed that *BCLX* splicing modulation showed no cytotoxic to normal astrocyte cells. Our findings confirmed aberrant *BCLX* mRNA splicing as a potential therapeutic target for GBM, and regulation of *BCLX* pre-mRNA splicing by SSOs is a novel approach to inhibit glioblastoma tumorigenesis alone or in combination with radiotherapy.

## Methods

### Public databases (TCGA, DepMap, GEO, CGGA)

The expression profiles of GSE50161 and GSE16011 were downloaded from the Gene Expression Omnibus (GEO) database. The catalog of gene essentiality across GBM cell lines is obtained from the Cancer Dependency Map Project (DepMap). RNA sequencing and clinical data were gathered from the UCSC (https://xenabrowser.net/) and CGGA (http://www.cgga.org.cn/index.jsp) databases. Bcl-xL expression profiles were analyzed based on the R Studio software.

### Cell culture and ionizing radiation

Human GBM cell lines A172, T98G, U251, U87, GSCs (Human glioma stem cell-like cells), and a normal human astrocyte cell line HA1800 were procured from ATCC (VA, USA). A172 cells were grown in RPMI-1640 medium containing 10% fetal bovine serum (FBS) (Ausbian, Australia). T98G, GSCs, and HA1800 were cultured in Dulbecco’s modified Eagle’s medium (DMEM) containing 10% FBS. U87 and U251 cells were grown in Minimum Essential Medium (MEM) containing 10% FBS. All cell lines were incubated at 37 °C and 5% CO_2_. Cells were exposed to the indicated doses of ionizing radiation using 225.0 KV X-rays from linear accelerators (X-RAD 225 OptiMax, PRECISION, USA) at a dose rate of 500 cGy/min.

### Splice-switching oligonucleotides transfection

Vivo-Morpholino modified oligonucleotides (vMO) and the specific vMO sequence labeled with fluorescein FAM were synthesized and purchased from Gene Tools (USA) and stored at room temperature. Stock vMO concentrations were calculated based on the A260. The oligonucleotide sequence 5′-GCTTGGTTCTTACCCAGCCGCCGTT-3′ (the underlined base being the splice joint), Bclx-vMO, was complementary to the junction of intron 2 and exon2b of *BCLX* pre-mRNA and the random scrambles sequence is cloned as: 5′-CCTCTTACCTCAGTTACAATTTATA-3′ (Rs-vMO). Cells were transfected with 2 μM, 4 μM, or 8 μM vMO as mentioned according to the manufacturer’s protocol and harvested 48 h post-transfection.

### Immunofluorescence microscopy

Cells were seeded and grown on Ψ20 mm glass-bottom cell culture dishes and fixed with 0.4% paraformaldehyde. Subsequently, cells were permeabilized in 0.5% Triton X-100, blocked in 10% BSA, and incubated overnight at 4 °C with primary antibodies. The following primary antibodies were tested: Bcl-xL (1:500, Abcam), SC35(1:200, Abcam). The cells were then stained with secondary antibodies and 4′,6-diamidino-2-phenylindole (Vector Laboratories, Burlingame, CA). Fluorescence images were visualized and captured using a confocal scanning microscope (LSM700, Zeiss, Germany).

### RNA extraction and RT-PCR

Cells were treated as indicated and RNA was isolated from cell pellets using RNeasy Mini Kit (Qiagen, Germany) following the manufacturer’s protocol. Reverse transcription was performed with the QuantiTect-Reverse-Transcription Kit (Qiagen, Germany) according to the manufacturer’s instructions. qRT-PCR was performed in triplicate using QuantiNova SYBR Green PCR Kit (QIAGEN, Germany) in QuanStudio 5 Real-time PCR system (Therom Lifetech ABI, USA). Primer sequences for qRT-PCR are listed in Supplementary Table 1.

### Immunoblotting

Cells lines were lysed using 1× RIPA Lysis (Solarbio, China) containing PMSF (1:100, Solarbio, China). Protein samples were quantified using the BCA protein assay kit (Solarbio, China) as manufacturer’s instructions. We loaded 20 μg protein per well into 10–12% gels and then transferred it to PVDF membranes using the Trans-Blot Turbo transfer system (Bio-Rad) according to the manufacturer’s instructions. Membranes were blocked for 1 h in 5% BSA and stained with primary antibody overnight at 4 °C. The following primary antibodies were tested: Bcl-xL (1:1000, Abcam), Bcl-xS (1:1000, GeneTex), *SQSTM1* (1:1000, Cell Signaling), LC3 (1:1000, Abcam), Beclin-1 (1:1000, Abcam), Cleaved-caspase3 (1:1000, Cell Signaling), Caspase3 (1:1000, Cell Signaling), Cytochrome c (1:1000, Immunoway). *BCL2* (1:1000, Proteintech), *MCL1* (1:1000, Proteintech). After washing the blots three times for 10 min each with TBST, the secondary antibody anti-rabbit horseradish peroxidase (1:10000, Immunoway) or anti-mouse horseradish peroxidase (1:10000, Immunoway) was added for 1 h at room temperature. Following another round of 3 × 10 min washes, the membranes were imaged on the Alpha Innotech followed by quantification using Image J.

### Cell viability assay

Viability of cells was determined using a 3-(4,5-dimethylthiazol-2-yl)-2,5-diphenyltetrazoliumbromide (MTT) assay. 6 × 10^3^ cells were plated in 96-well plates for 24 h of incubation for treatment with vMO. 48 h after treatment, MTT was added to each well for incubation for 1 h at 37 °C. Absorbance at 490 nm was determined for each well using multifunction microplate reader (Tecan Infinite 200 M, Männedorf Switzerland).

### Cytotoxicity assay

Cells were seeded into a 96-well plate and treated with vMO. The ApoTox-Glo™ Triplex Assay kit (Promega Corporation, WI, USA) was used to simultaneously measures two protease activities. One is a marker of cell viability, and the other is a marker of cytotoxicity. The assay was performed according to the manufacturer’s instructions.

### Mitochondrial membrane potential (MMP) assays

Cells were seeded into a 96-well plate (Corning) and treated with vMO for 48 h. Next, cells were stained with JC-10 solution for 30 min and photographed using the high content analysis system (PerkinElmer, MA, USA).

### Flow cytometry analysis of apoptosis

Annexin V/PI apoptosis kit (MULTISCIENCES, China) was used to detect the apoptosis rate of each sample. Cells were cultured in six-well plates (2–3 × 10^5^ cells/well) for 24 h before treatment and collected at the end of the indicated treatment. Each sample was resuspended in 150 μl binding buffer, screened, and incubated with 5 μl Annexin V-FITC and 10 μl PI fluorescent dyes. The apoptosis rate was detected using a flow cytometer and analyzed with Flowjo V10 software.

### Three-dimensional (3D) microsphere model

A172 cells (3–4 × 10^3^ cells per well) were seeded in U-bottom 3D cell culture plates (PerkinElmer, spheroid ULA/CS, CellCarrier-96) and cultured for 24 h to form a three-dimensional structure. After the microspheres became visible to the naked eye, cells were treated as indicated and analyzed with a Nikon Eclipse Ti inverted microscope (Nikon, Tokyo, Japan). The viability of spheroid cells was marked by SYTOX Green as indicated (Alphabio, China) and captured by a confocal scanning microscope (Carl Zeiss, Jena, Germany).

### PCR array assay

QuantiNova LNA PCR Focus panels (Qiagen, Germany) in 96-well plates were used to measure the expression of genes associated with signaling pathways. The panels contain assays for 84 apoptosis pathway-focused genes and 5 reference genes were detected according to the manufacturer’s instructions.

### Autophagosomes detection

Cells were seeded and grown on Ψ20mm glass-bottom cell culture dishes and treated as indicated. Next, the cells were stained with Cell Meter™ Autophagy Assay Kit (AAT Bioquest, CA, US) and Hoechst 33342 (Meilunbio, China) based on the manufacturer’s instructions. The images were visualized by a confocal scanning microscope and analyzed by Image J.

### Transmission electron microscopy (TEM)

A172 cells were trypsinized and pelleted. The samples were then fixed with Fixative for TEM (Servicebio, Wuhan, China) for 2–4 h at 4 °C. The 0.1 M PB (pH 7.4) was added into the tube after supernatant was discarded to re-suspend and wash. The 1% agarose solution was prepared for the pre-embedding. Agarose blocks with samples avoid light post-fixed with 1% OsO_4_ (Ted Pella Inc, CA, US) in 0.1 M PB (pH 7.4) for 2 h at room temperature. After being rinsed in 0.1 M PB (pH 7.4), the cells were dehydrated at room temperature and embedded by resin. The resin blocks were cut to 60–80 nm thin on the ultramicrotome (Leica UC7, Wetzlar, Germany) and stained 2% uranium acetate and 2.6% lead citrate. The cuprum grids are observed under TEM (Hitachi, Tokyo, Japan) and take images.

### 5-Ethynyl-2′-deoxyuridine (EdU) assay

DNA synthesis in cells was detected using the EdU-555 kit (Meilunbio, China) according to the reagent instructions. Images were captured and counted in ≥seven randomly chosen visual fields under a microscope confocal scanning microscope.

### Colony-forming assay

After irradiation, the cells were trypsinized and added to a 6-well culture plate at a density of 2 × 10^3^ in complete medium. Being incubated for 10–14 days at 37 °C, the plated cells were fixed with 4% paraformaldehyde and dyed with 1% crystal violet. Colonies (≥50 cells as a colony) were counted under a dissecting microscope. The surviving fraction (SF) of the treated group was calculated as SF = colonies counted / (cells seeded x PE) and revised via linear-quadratic equation.

### Statistical analysis

Statistical analysis was performed using GraphPad Prism. Data represent biological replicates (*n* ≥ 3) and are depicted as mean values ± S.D. as indicated in the figure legends. The statistical analyses performed for different data are demonstrated in each figure legend, and the data is considered statistically significant if *p* < 0.05.

## Results

### Bcl-xL variant is markedly overexpressed in human GBM and correlated with poor prognosis

Differentially spliced isoforms caused by deregulated splicing profiles are important contributors to tumorigenesis and treatment resistance [27]. Investigating the RNA-Seq from GEO (GSE16011, GSE50161), we found that comparing GBM with the normal brain, splicing pathways were significantly upregulated (Supplementary Fig. 1A–D). Splicing aberrations are caused by dysregulated expression of proteins involved in alternative splicing, often resulting in morphology disturbances of nuclear speckles. We therefore observed the organization of splicing factors within the nucleus. Splicing factors accumulated to much larger structures in the nucleus of GBM cells compared to normal brain cells, indicating that abnormal splicing event may be a general phenomenon in GBM (Supplementary Fig. 1E).

**Fig. 1:**
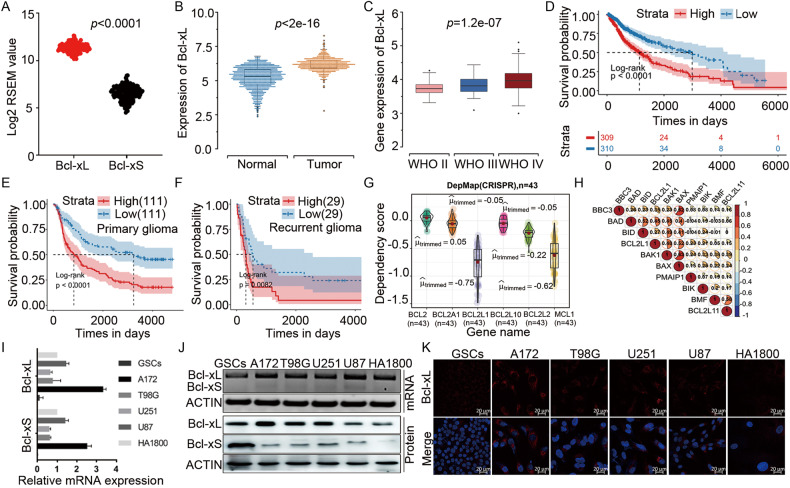
Anti-apoptotic Bcl-xL is highly expressed in GBM samples and GBM cell lines, and correlated with worse prognosis. **A** Comparison of Bcl-xL and Bcl-xS mRNA expression levels between GBM tissues based on TSVdb (http://tsvdb.com/index.html) database. **B** Expression of Bcl-xL in normal brains and GBM from TCGA. **C** Correlation between Bcl-xL expression and pathologic grades of GBM. **D**–**F** Kaplan–Meier survival analysis for Bcl-xL variant expression in patients with GBM from TCGA and CGGA datasets. **G** The dependency profile of pro-survival *BCL2* family genes (*BCL2L1, BCL2L10, BCL2, BCL2A1, BCL2L2, and MCL1*) in GBM cell lines based on genome-wide CRISPR. Data were downloaded from the DepMap datasets (DepMap; https://depmap.org/portal/). **H** Correlation analysis of the expression of pro-apoptotic *BCL2* family genes and anti-apoptotic *BCL2* family genes from TCGA datasets. **I** The mRNA expression of Bcl-xL and Bcl-xS were analyzed by q-PCR. **J** The expression of *BCLX* splicing isoforms in GBM cell lines and HA1800 cells were analyzed by RT-PCR and Western Blot. **K** Immunofluorescence staining of Bcl-xL protein in GBM cells and HA1800 cells. Data in vitro represent at least three independent experiments. Anti-apoptotic Bcl-xL is highly expressed in GBM samples and GBM cell lines, and correlated with worse prognosis.

*BCLX* gene is alternatively spliced to generate anti-apoptotic Bcl-xL and pro-apoptotic Bcl-xS. The preferential inclusion of *BCLX* exon2b (Bcl-xL) was detected in many cancers and considered a general mechanism by which cancer cells evade apoptosis [3]. We first investigated the exon expression of Bcl-xL and Bcl-xS based on TSVdb database. The result showed that anti-apoptotic Bcl-xL had a high expression in GBM samples (Fig. 1A). Consistently, Bcl-xL expression is significantly upregulated in patients’ GBM compared to normal brain tissues in TCGA-dataset (Fig. 1B). A marked Bcl-xL overexpression is associated with the clinical stage of GBM (CGGA-dataset) and worse survival for both primary and recurrent brain tumors (CGGA-dataset, TCGA-dataset) (Fig. 1C–F). We also investigated the dependency profile of *BCL2* family proteins (*BCL2*, *BCL2L10*, *BCL2A1*, *BCL2L1*, and *MCL1*) in GBM within Cancer Cell Line Encyclopedia, and found that GBM cells show the greatest dependency on Bcl-xL (*BCL2L1*) for survival (Fig. 1G). Moreover, highly expressed Bcl-xL (*BCL2L1*) is positively associated with pro-apoptotic *BCL2* proteins (Fig. 1H), supporting the theory that GBM is primed for apoptotic death [28, 29]. We also determined the splicing and expression profile of *BCLX* in GBM cell lines and a normal astrocyte cells HA1800 by RT-PCR, western blot, and immunofluorescence assays. The results showed that anti-apoptotic Bcl-xL is preferentially spliced in all cell types, especially in A172 and U87 cells. However, at the protein level, Bcl-xL expression was higher in GBM cell lines than in normal astrocyte cells (Fig. 1I–K). Taken together, dysregulation of *BCLX* apoptotic isoforms caused by precarious equilibrium splicing is implicated in the development and progression of GBM.

### Bclx-vMO transfection could correct the abnormal *BCLX* splicing in GBM cells

**Fig. 2:**
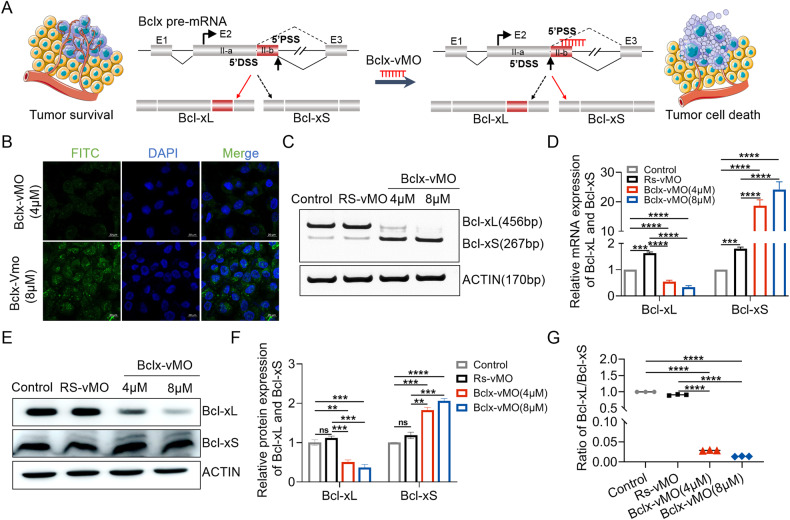
Bclx-vMO regulated Bcl-x splicing mode in GBM cells effectively. **A** Alternative splicing mode and splicing regulation by SSOs in cancer cells. **B** Localization and delivery efficiencies of Bclx-vMO using Laser confocal microscopy. Fluorescence signal represented the Bclx-vMO delivered by Endo-Porter combined to the target RNA and increased with the amount of Bclx-vMO used. **C**–**F** A172 cells were transfected with 4, 8, μM vMO. The expression of Bcl-xL and Bcl-xS were analyzed by PCR and western blot. Bclx-vMO elevated Bcl-xS at the expense of Bcl- xL both at mRNA (**C**, **D**) and protein levels (**E**, **F**), and significantly decreased the ratio of Bcl-xL/Bcl-xS. **G** Data are shown as mean values ± S.D. from three independent experiments. One-Way ANOVA followed by Dunnett’s multiple comparisons test are reported. For all panels “**” indicates *p* < 0.01, “***” indicates *p* < 0.001, “****” indicates *p* < 0.0001, “ns” indicates no significance.

SSOs, typically 15–20 nucleotides, is a kind of synthetic and steric block antisense oligonucleotides which have been widely used to disrupt the splicing mode of pre-mRNA through Watson-Crick base pairing. SSOs targeting *BCLX* pre-mRNA might able to shift dysregulated *BCLX* splicing towards its pro-apoptotic variant Bcl-xS and result in sensitized cell death in cancer (Fig. 2A). A172 cells were transfected with vivo-Morpholino modified oligonucleotides (Bclx-vMO), which targeted the downstream 5′ alternative splice site of exon 2. The results displayed that 48 h after transfection, the dim and diffuse fluorescence throughout the cell could be observed, indicating successful delivery (Fig. 2B). To further verify whether vMO can correct *BCLX* splicing, the experiments were divided into three groups: scramble sequence vMO (RS-vMO) group, Bclx-vMO group, and no-treatment control group. RT-PCR confirmed that Bclx-vMO could effectively increase Bcl-xS mRNA at the expense of Bcl-xL mRNA (Fig. 2C, D). The same results for protein expression were obtained by western blot analysis (Fig. 2E, F). These results demonstrated that *BCLX*-specific SSOs allowed Bcl-xL to switch to Bcl-xS, efficiently reversed the abnormal splicing of BCLX gene in A172 cells (Fig. 2G), and can be used as a potential therapeutic target for GBM.

### Bclx-vMO inhibited the proliferation and promoted apoptosis of GBM cells in vitro but not in normal astrocyte cells

**Fig. 3:**
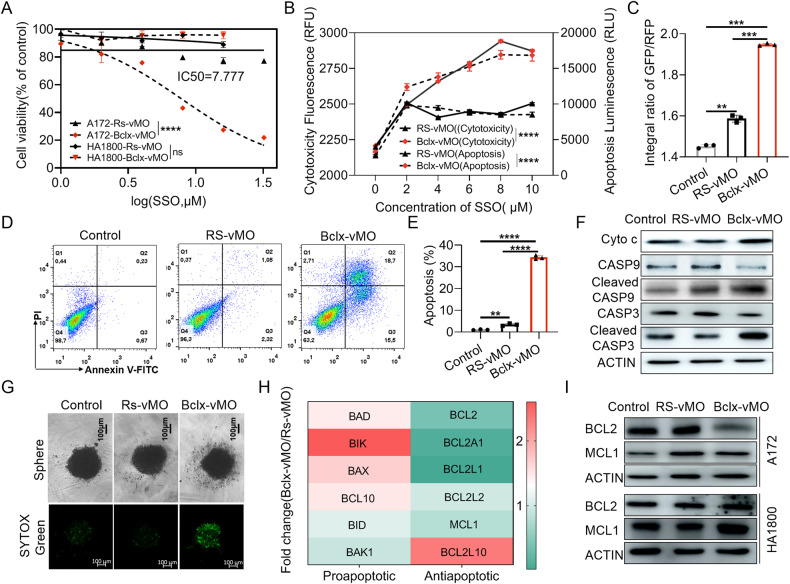
Cytotoxicity of GBM cell lines after shift of *Bcl-x* pre-mRNA alternative splicing from Bcl-xL to Bcl-xS. **A** The cell viability of different concentrations of Rs-vMO and Bclx-vMO on A172 cells and normal astrocyte HA1800 after 48 h transfection (Two-way Anova). **B** Cell cytotoxicity and apoptosis assay of A172 cells after 48 h transfection (Two-way Anova). **C** Fluorescence of A172 cells stained using JC-10 was ascertained through high content analysis system. **D**, **E** Apoptosis rate of A172 cell lines received 4 μM doses of vMO were detected by flow cytometer. **F** Western blot analysis of activated apoptin expression in A172 cell lines treated with vMO for 48 h. **G** 3D Cell viability of A172 cells treated with vMO was measured using SYTOX green and images were visualized and captured using optical microscope and confocal scanning microscope. **H** PCR array analysis of pro-apoptotic and anti-apoptotic *BCL2* family members. **I** Western blot analysis of anti-apoptotic BCL2 family protein (*BCL2, MCL1*) expression in A172 cell lines treated with vMO for 48 h. The concentration of Rs-vMO and Bclx-vMO used was 4 μM when not specified. Data are shown as mean values ± S.D. from three independent experiments. One-Way ANOVA followed by Dunnett’s multiple comparisons test are reported. For all panels, “***” indicates *p* < 0.001, “****” indicates *p* < 0.0001.

A cell viability assay was performed to examine the effect of Bclx-vMO on GBM cells (A172) and normal astrocyte cells (HA1800). Bclx-vMO-mediated splice switch markedly inhibited the proliferation of A172 cells but showed no toxicity in normal astrocyte cells, suggesting that Bcl-xL is essential for GBM cell survival (Fig. 3A). In addition, Bclx-vMO treatment showed significant cytotoxicity and active caspase 3/7 activity compared with cells treated with Rs-vMO (Fig. 3B). Caspases 3/7 activity has been proposed as critical mediators of mitochondrial pathways of apoptosis [30]. Therefore, we determined the mitochondrial damage by JC-10 staining. Bclx-vMO treatment showed an increased ratio of GFP/RFP compared with control groups, indicating impaired MMP (Fig. 3C, Supplementary Fig. 2A). Consistently, flow cytometry analysis revealed that *BCLX* splicing regulation with Bclx-vMO led to pronounced apoptosis of A172 cells at 48 h post-transfection (Fig. 3D, E). Furthermore, immunoblotting analysis of the CASP9/CASP3 cleavage fragment had also confirmed that Bclx-vMO markedly induced CASP9/CASP3 activation (Fig. 3F, Supplementary Fig. 2B). Compared with 2D cell culture, 3D cell culture provides more valuable information about cellular communication and clinically representative response to therapeutic agents [31]. We established 3D-A172 cell microspheres model and investigated the pro-apoptotic effect of Bclx-vMO by SYTOX Green. The results demonstrated that Bclx-vMO treatment reduced the size of 3D-A172 cell microspheres and lead to significantly increased apoptosis (Fig. 3G, Supplementary Fig. 2C). However, there was little cytotoxicity in MMP and apoptosis of human normal astrocyte cells treated with Bclx-vMO (Supplementary Fig. 3).

*BCL2* family of anti- and pro-apoptotic proteins controlled the balance between survival versus death of cells, we further accessed the effect of Bclx-vMO on the mRNA expression of *BCL2* family by PCR array. The shift in *BCLX* splicing to Bcl-xS stimulated the expression of pro-apoptotic *BCL2* genes (*BIK, BAX, BAD, BCL10*) but inhibited the anti-apoptotic *BCL2* genes (*BCL2, BCL2A1, BCL2L11, MCL1*) on the arrays (Fig. 3H), indicating that Bcl-xL may play an important role in regulating the balance of *BCL2* family proteins and determining the apoptotic baseline of A172 cells. Furthermore, given that normal astrocyte cells demonstrated significant resistance to the Bcl-xL splicing inhibition, we speculated whether Bcl-xL inhibition cause a compensatory elevation of other anti-apoptotic *BCL2* family proteins in HA1800. The expression of anti-apoptotic *BCL2* and *MCL1* in A172 and HA1800 cells were detected. The result showed that *BCL2* expression in normal astrocyte cells did not change compared with A172, while *MCL1* expression was upregulated after Bclx-vMO treatment (Fig. 3I, Supplementary Fig. 2D, E), suggesting that MCL1 may play a pro-survival role in HA1800 cells after Bcl-xL inhibition. Taken together, these data indicated that A172 cells were Bcl-xL-dependent and shift *BCLX* splicing to pro-apoptotic Bcl-xS induced markedly toxicity both in 2D and 3D-A172 cell cultures but not in normal astrocyte cells.

### The splice shift induced by Bclx-vMO promoted autophagic flux

**Fig. 4:**
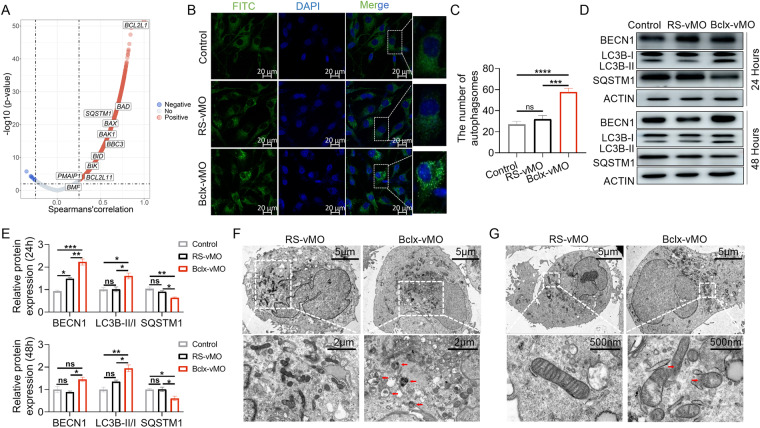
Correction of *BCLX* pre-mRNA alternative splicing from Bcl-xL to Bcl-xS induces autophagy in A172 cancer cells. **A** Volcano plot of Spearmans’ rank correlation coefficient between Bcl-xL (*BCL2L1*) and tested proteins. Gene expression data were downloaded from TCGA-GBM. The “*corrplot*” package in R was used to obtain the correlation coefficient and *p*-value of genes correlated with Bcl-xL expression. **B**, **C** The autophagosomes induced by Bclx-vMO were visualized and quantified. **D**, **E** The expression of BECN1, SQSTM1/p62, and conversion of LC3I to LC3II were detected and analyzed through western blotting. **F**, **G** A172 cells were treated with 4 μM Bclx-vMO for 48 h. Representative microscopy images were obtained by transmission electron microscopy. The red arrow indicates autophagic vacuoles containing cytoplasmic context (**F**) or damaged mitochondrial membranes (**G**). The concentration of Rs-vMO and Bclx-vMO used was 4 μM when not specified. Data are shown as mean values ± S.D. from three independent experiments. One-Way ANOVA followed by Dunnett’s multiple comparisons test are reported. For all panels “*” indicates *p* < 0.05, “****” indicates *p* < 0.0001, “ns” indicates no significance.

Autophagy is associated with the maintenance of cell homeostasis and has received tremendous attention in cancer research. Due to the opposing roles and context-dependent effects of autophagy within oncology, approaches with interventions designed to inhibit or enhance autophagy in cancer therapy will not be successful [32]. Many of the current treatments themselves affect autophagy. Spearman’s correlation between gene expression levels in the TCGA-GBM dataset was calculated using the “*corrplot*” package and the results revealed that *SQSTM1*/p62, an indicator of autophagy, is positively correlated with Bcl-xL (Fig. 4A). We next examined whether modulation of *BCLX* splicing induces autophagy flux in GBM. Autophagy Assay Kit staining revealed a significant increase in the number of autophagosomes in A172 cells treated with Bclx-vMO (Fig. 4B, C). It is generally accepted that the conversion of LC3I to LC3II concomitant with the consumption of *SQSTM1*/p62 is a hallmark of increased autophagy flux [33]. We found that LC3I had converted to LC3II and p62 expression was reduced after the splicing modulation of *BCLX* for 24 and 48 h (Fig. 4D, E). Furthermore, transmission electron microscopy (TEM) was used to observe ultrastructural changes in A172 cells treated with Bclx-vMO. The TEM studies revealed that Bclx-vMO treated GBM cells acquired a significantly higher number of autophagic vacuoles in comparison to Rs-vMO (Fig. 4F), and a large number of myelinfigures was also found in lysosomes. Additionally, our results also showed mitochondrial swelling accompanied by dissipation of cristae structures and mitochondrial membrane damage in Bclx-vMO treated A172 cells (Fig. 4G). No significant change in autophagic flux was detected in normal astrocyte cells (Supplementary Fig. 4). Overall, these data indicated that the splicing correction of *BCLX* gene by Bclx-vMO could also activate autophagy in GBM cells.

### Bclx-vMO induced autophagy is an apoptosis-linked and tumor suppressor process in GBM cells

**Fig. 5:**
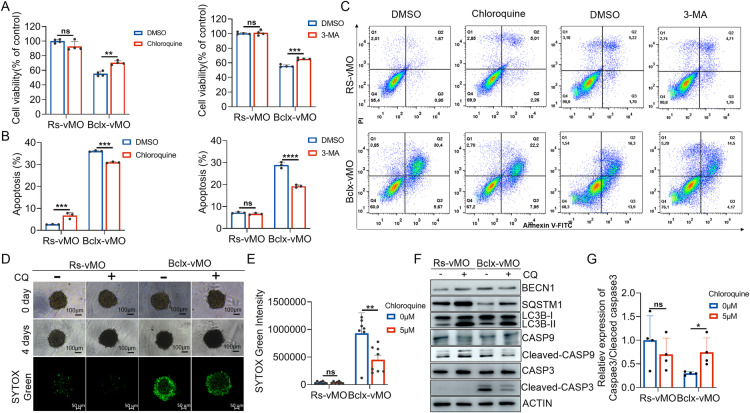
Blocking *BCLX* splicing regulation-induced autophagy significantly attenuates apoptosis in A172 cells. **A** A172 cells were co-incubated with CQ or 3-MA and Bclx-vMO for 48 h. Cell viability was assessed by MTT assay. **B**, **C** A172 cells were co-incubated with CQ or 3-MA and Bclx-vMO for 48 h. Then, the apoptosis rate of A172 cells detected by flow cytometer. Quantification of apoptosis in cells treated is presented. **D**, **E** 3D Cell viability of A172 combined-treated with Bclx-vMO and CQ was measured using SYTOX Green. **F**, **G** Total proteins of A172 cells were extracted and the expression of proteins related to apoptosis and autophagy was detected by western blot. The concentration of Rs-vMO and Bclx-vMO used was 4 μM when not specified. Data are shown as mean values ± S.D. from ≥three independent experiments. One-Way ANOVA followed by Dunnett’s multiple comparisons test are reported. For all panels “*” indicates *p* < 0.05, “**” indicates *p* < 0.01, “***” indicates *p* < 0.001, “****” indicates *p* < 0.0001, “ns” indicates no significance.

Because Bclx-vMO treatment induced caspase-dependent apoptosis and autophagy in A172 cells simultaneously, these findings led us to investigate the functional crosstalk between autophagy and apoptosis induced by *BCLX* splicing regulation. We investigated whether Bclx-vMO-induced autophagy is an independent or apoptosis-linked process. We first blocked Bclx-vMO-induced autophagy with CQ or 3-MA in A172 cells. As shown in Fig. 5A, compared with the Bclx-vMO treatment alone, the combined treatment with CQ or 3-MA partially attenuated the cytotoxic effect of *BCLX* splicing modulation. In accordance with the antiproliferative activity, results of flow cytometric assays confirmed that combined treatment with CQ or 3-MA markedly attenuated Bclx-vMO-induced cell apoptosis (Fig. 5B, C). In addition, the 3D-A172 cell microsphere model showed that CQ could decrease the proportion of dead cells induced by Bclx-vMO (Fig. 5D, E), indicating that the autophagy enhanced the antiproliferative effects and apoptosis in Bclx-vMO treated cells. Furthermore, western blotting analysis showed that the cleavage of CASP3 was inhibited by CQ compared to cells treated with Bclx-vMO alone (Fig. 5F, G). Collectively, these data showed that Bcl-xL splicing inhibition-induced autophagy could promoted apoptosis induction in A172 cells, which is in line with the role of autophagy induced by BH3 mimetics in cancer cells [34, 35].

### X-ray radiation-induced anti-apoptotic Bcl-xL expression

**Fig. 6:**
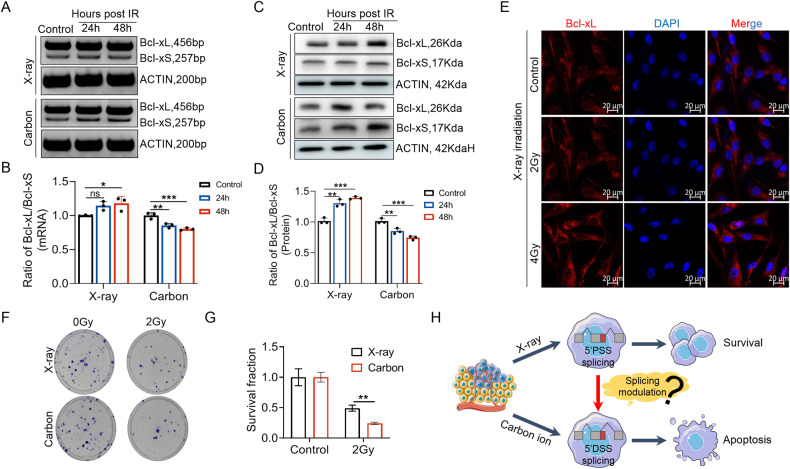
Effect of X-ray and heavy ion irradiation on the alternative splicing and expression of *BCLX* gene. **A**, **B** The splicing patterns of *BCLX* gene after X-ray and carbon ion irradiation was assessed by RT-PCR analysis. **C**, **D** Effect of X-ray and carbon ion irradiation on the expression of Bcl-xL and Bcl-xS at protein level. **E** Effect of X-ray irradiation on the spatial distribution and expression of Bcl-xL isoforms. **F**, **G** Clonal survival of A172 cells after X-ray and carbon ion irradiation. **H** Schematic diagram of the different splicing patterns of *BCLX* after X-ray and carbon ion irradiation. Data are shown as mean values ± S.D. from three independent experiments. One-way ANOVA followed by Dunnett’s multiple comparisons test are reported. For all panels “*” indicates *p* < 0.05, “**” indicates *p* < 0.01, “***” indicates *p* < 0.001, “ns” indicates no significance.

Radiotherapy is a current standard-of-care treatment and is used widely for GBM. Although GBM patients initially respond to radiotherapy, the development of adaptive radioresistance of several GBM cells often results in inevitable recurrence [36]. Studies have shown that anti-apoptotic proteins of *BCL2* family, including Bcl-xL and *BCL2*, play an important role in the acquisition of radiation resistance in certain tumor types [21, 37]. We investigated if radiation could affect the splicing patterns of *BCLX* gene in GBM cells. The expression of *BCLX* splice isoforms was assessed by RT-PCR and q-PCR (Fig. 6A, Supplementary Fig. 5). Compared with carbon ion radiation, A172 cells irradiated with low energy X-rays induced an increased ratio of Bcl-xL / Bcl-xS after irradiation (Fig. 6B, Supplementary Fig. 5). We also detected the protein expression of Bcl-xL and Bcl-xS. Similar results were observed that X-ray irradiation increased the ratio of Bcl-xL / Bcl-xS while carbon ion radiation decreased it (Fig. 6C, D). Immunofluorescence results also showed that the Bcl-xL expression in the cytoplasm was significantly increased 48 h after X-ray radiation (Fig. 6E). Compared to conventional proton irradiation, carbon ion radiotherapy induced a stronger lethal effect on cancer cells due to the specific radiobiological features [38]. Colony formation assay indicated that carbon ion-induced a stronger inhibitory effect than X-rays in A172 cells after radiation (Fig. 6F, G). Thus, we speculated that carbon ion radiation might promote the splicing of pro-apoptotic Bcl-xS, while X-rays might promote the expression of anti-apoptotic Bcl-xL to benefit radioresistance. These results implied that Bcl-xL-favored splicing may play a crucial role in tumor progression and radioresistance development of A172 cells, it also implied that the combination of X-ray radiotherapy and *BCLX* splicing modulation may effectively enhance the radiosensitivity of tumor cells (Fig. 6H).

### Modulation of *BCLX* alternative splicing renders GBM cells more susceptible to radiation

**Fig. 7:**
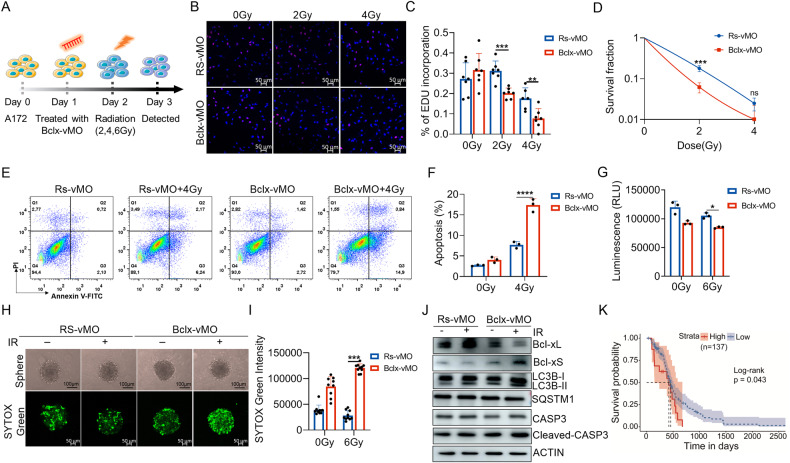
Correction of *BCLX* splicing from anti-apoptotic Bcl-xL to Bcl-xS sensitizes GBM cells to IR. **A** Schematic illustration of experiment design and treatment plan. **B**, **C** EdU assay demonstrated that Bclx-vMO could sensitize A172 cells to X-ray irradiation. Typical photos of the EdU assay were captured with confocal microscopy. **D** Colony formation assays using A172 cells with inhibition of Bcl-xL or control combined with or without IR treatment. **E**, **F** Apoptosis of A172 cells with *BCLX* splicing correction or control combined with or without IR were evaluated by flow cytometry. **G** The cell viability of 3D spheroid cultures of A172 cells was determined using Cell Titer-Glo 3D according to the instructions. **H**, **I** The dead cells in 3D spheroid cultures of A172 with *BCLX* splicing correction or control combined with or without IR were measured using SYTOX Green. **J** Western blot was performed to measure the levels of proteins related to apoptosis and autophagy in A172 cells with splicing modulation upon IR. **K** Kaplan–Meier survival analysis of GBM patients who received radiation based on Bcl-xL expression (TCGA datasets). Data are shown as mean values ± S.D. from ≥three independent experiments. One-way ANOVA followed by Dunnett’s multiple comparisons test are reported. For all panels “***” indicates *p* < 0.001, “****” indicates *p* < 0.0001.

Since X-ray radiation-induced increased expression of anti-apoptotic Bcl-xL [29], we hypothesized that inhibiting the upregulated Bcl-xL by Bclx-vMO may sensitize GBM cells to radiation. To determine if Bclx-vMO could restore the radiosensitivity of A172 cells, both the nonradiated cells and cells subjected to 2–4 Gy radiation were treated with Bclx-vMO with a Bcl-xL inhibition rate of about 50% (Fig. 7A, Supplementary Fig. 6A). Subsequently, cell proliferation measured by EdU assay showed that Bclx-vMO treatment significantly sensitized A172 cells to X-ray radiation (Fig. 7B, C). The survival curves under radiation also indicated that Bcl-xL inhibition could increase radiosensitivity in A172 cells (Fig. 7D). Besides, ionizing radiation induces apoptosis in cancer cells. We found that Bclx-vMO combined with X-ray radiation, harbored significantly better apoptosis rate than single radiation (Fig. 7E, F, Supplementary Fig. 6B, C). In addition, three-dimensional cell cultures have been used to evaluate the efficacy of radiotherapy because they are easy to cultivate and can mimic tumors in vivo [39]. It has been reported that the effect of X-ray irradiation was attenuated in 3D spheroid cultures [39]. Therefore, we used a 3D spheroid model to further explore the radiosensitization activity of Bclx-vMO. Cell viability of 3D spheroid cultures was determined using a Cell Titer-Glo assay. The result showed that 6 Gy X-ray radiation combination with Bclx-vMO reduced the viability of the 3D cell cultures than radiation alone (Fig. 7G). We further used SYTOX to determine the dead cells in 3D spheroid cultures and found an obvious cell death in the combinational group (Fig. 7H, I). Furthermore, we found Bclx-vMO combined with radiation significantly reduced X-ray-induced Bcl-xL expression, but increased expression of pro-apoptotic Bcl-xS and activated CASP3 (Fig. 7J). Additionally, the results showed that Bclx-vMO treatment also sensitizes A172 cells to carbon ion radiation (Supplementary Fig. 7A–D). To further support the radiosensitizing effect of Bcl-xL inhibition, we utilized the selective inhibitor of Bcl-xL WEHI-539 in combination with X-ray treatment on A172 cells. The results showed that low-dose WEHI-539 treatment significantly increased the radiosensitivity of A172 cells, and inhibit proliferation and enhance apoptosis (Supplementary Fig. 8). At the clinical level, we verified the roles of Bcl-xL in radiation treatment response using samples from GBM patients. The result showed that patients with a high Bcl-xL expression harbored a significantly shorter survival time during radiotherapy (Fig. 7K). These data suggested that Bclx-vMO caused splicing correction of *BCLX* lead to radiosensitization in A172 cells compared with radiation alone and may be an effective treatment for combination therapy of GBM.

## Discussion

Pre-mRNA splicing is essential for the expression of >95% human genes and is a main mechanism used to regulate cellular homeostasis and development. Abnormalities of AS have been increasingly implicated in the development of multiple tumors [40–43]. Cancer-specific alterations in splicing process could contribute to every hallmark of tumor, including oncogenesis, immune responses, as well as evade to anti-cancer therapy [27, 44–47]. The preferentially expressed isoforms that could promote growth and invasion in both primary and recurrent GBM have been identified successively [48, 49], suggesting that RNA splicing dysregulation may be a general glioblastoma characteristics. Using the RNA-Seq analysis from GEO comparing GBM with the normal brain, we showed that the splicing pathways were upregulated in GBM tissues, implying that GBM may be prone to disrupted alternative splicing and exhibit a more complex splicing repertoire than normal tissues.

Multiple genes that control cell death are regulated at the splicing level, especially *BCL2* family members. *BCL2* family has been identified for its role in apoptosis regulation. The members of *BCL2* family are designated as pro-survival subgroups (*BCL2*, Bcl-xL*, BCLW, MCL1, A1/Bcl-B*) that promotes cell survival, pro-apoptotic BAX/BAK-like proteins act as effectors of apoptosis, and BH3-only proteins (*BIM, PUMA, BID, NOXA, BMF, BIK*, and *HRK*) to initiate apoptosis [50, 51]. Most of the *BCL2* family members have two or more splicing variants that provide functional diversification to the control of apoptosis. *BCLX* gene has 44% homology to *BCL2* and generates two major isoforms, anti-apoptotic Bcl-xL and pro-apoptotic Bcl-xS [15, 52]. The favored splicing of anti-apoptotic Bcl-xL has been found implicated in genesis and development of Hodgkin lymphoma [53], colorectal cancer [54]. Overexpressed Bcl-xL also had been suggested to be involved in metastasis, drug- and radioresistance of multiple cancer types [15]. In this study, we identified the favored spliced Bcl-xL in GBM cell types. Highly expressed Bcl-xL is positively correlated with the clinical stage but negatively correlated with the prognosis of GBM patients. Interestingly, we also found that GBM have a higher survival dependence on Bcl-xL compared to other pro-survival proteins of *BCL2* family. These findings suggest targeting aberrant splicing of Bcl-xL may provide a promising therapeutic regimen for the treatment of GBM.

AS modulation can be achieved by regulating the activity of key splicing factors (broad-spectrum) or by precisely targeting specific isoforms [2]. Multiple small molecular compounds that target splicing factors and affect global splicing have been identified over the years and showed preclinical promise [2, 8, 27, 55]. However, many disease-related splicing factors are not druggable and lack of selectivity. Splice-switching oligonucleotides (SSOs) are short, modified oligos and have been used to modulate aberrant splicing by targeting 5′SS/3′SS or splicing enhancers or silencers on pre-mRNA sequence. SSOs drugs have been approved for the treatment of SMA [56] and DMD [57]. However, the development of SSOs drugs in oncology has lagged behind that in genetic diseases. We designed the splice-switching vivo-morpholino oligonucleotides targeting 5′SS of exon 2 in *BCLX* pre-mRNA. Bclx-vMO could simultaneously reduce Bcl-xL and increase Bcl-xS expression to promote apoptosis in 2D and 3D GBM cell models, but showed little cytotoxicity in normal astrocyte cells. Furthermore, *BCLX* splicing correction also promoted autophagy flux in GBM cells, and inhibition of autophagy reduced the level of apoptosis. Therefore, we speculated that autophagy mediated by Bclx-vMO may promote the execution of apoptotic functions when apoptosis occurs in large amounts.

Although aberrant AS is a common hallmark in tumors, few studies focus on the splicing changes during radiotherapy. It has been reported that UV irradiation could regulate AS by inhibiting RNA polymerase II elongation [58], suggesting that transcriptional coupling to AS is a key feature of the DNA-damage response. In addition, SRSF1, a crucial family member of SR proteins and promotes Bcl-xL splicing [59], has been reported to show an elevated expression after ionizing radiation in H1299 and A549 cells [7]. These results suggest that radiation may regulate AS decisions of downstream genes by affecting the coupling between transcript and splicing or regulating the expression of splicing factors. We detected different splicing patterns after different LET irradiation of the *BCLX* gene. The result showed that High-LET carbon ion irradiation can promote pro-apoptotic Bcl-xS splicing decisions, whereas low-LET X-rays promoted anti-apoptotic Bcl-xL splicing and might confer radioresistances. The specific mechanisms by which ion irradiation regulates AS events need to be further studied. In addition, since the complex crosstalk of *BCL2* family genes, it would be highly informative to assess the impact of ionizing radiation on alternative splicing of the other members of the *BCL2* family, in order to see whether there is a combined effect.

Bclx-vMO reduced the ratio of Bcl-xL/Bcl-xS and made cells primed-to-apoptosis, we hypothesized that pretreatment of cells with Bclx-vMO may amplify the radiation-induced apoptotic stimulus. Consistently, our results confirmed the radiosensitizing effect of Bclx-vMO treatment in A172 cells. In addition, the Bcl-xL protein inhibitor WEHI-539 also showed a radiosensitizing effect. Despite selectivity, it still not fully met the major criteria for defining a bona fide BH3 mimetic as proposed by Lessene et.al. [60]. The off-target effects and acquired resistance to BH3 mimetics are still important clinical problem [61, 62]. In addition, conventional treatments generally induce transient therapeutic effects because they target proteins rather than underlying causes. In contrast, nucleic acid therapeutics such as SSOs can achieve long-lasting or even curative effects via gene inhibition or editing [63]. The clinical translation, however, depends on delivery technologies that facilitate internalization and increase target affinity [63, 64].

## Conclusions

**Fig. 8:**
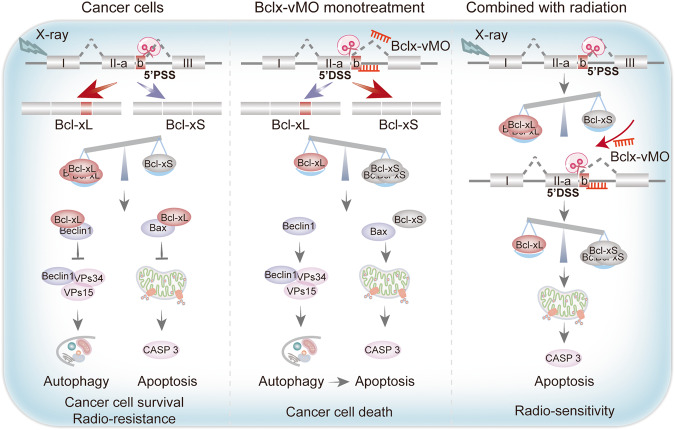
Schematic representation showing the proposed mechanisms through which the *BCLX* splicing modulation regulates apoptosis and radiosensitivity in GBM cells.

In conclusion, our study revealed that aberrant splicing may be prevalent in GBM. Bcl-xL isoforms are preferentially spliced over Bcl-xS in GBM and negatively correlated with prognosis. Low-LET irradiation can further enhance this splice selection and affect the radiosensitivity of GBM. SSOs-based splicing correction can promote the death of GBM cells and significantly enhance the radiosensitivity (Fig. 8). These studies provide a rationale for targeting splicing correction of specific oncogenes by SSOs. It also paves the way for future clinical trials of targeted splice-switching oligonucleotide drugs in combination with radiotherapy.

### Supplementary information

Supplementary materials

Original Data File

corResult of BCL2L1.csv

## Data Availability

The datasets generated during and/or analyzed during the current study are not publicly available but are available from the corresponding author on reasonable request.
