# Supplementary material for: Modification of BCLX pre-mRNA splicing has antitumor efficacy alone or in combination with radiotherapy in human glioblastoma cells
Source: Cell Death Dis. 2024 Feb 21;15(2):160. doi: 10.1038/s41419-024-06507-x (PMC10881996; doi:10.1038/s41419-024-06507-x)
Supplement: Supplementary file 1 — Supplementary materials [file 41419_2024_6507_MOESM1_ESM.docx]

**Modification of *BCLX* pre-mRNA splicing has antitumor efficacy alone or in combination with radiotherapy in human glioblastoma cells**

**Supplemental Table 1. Primer sequences**

| Bcl-x Forward | 5'- AGTAAAGCAAGCGCTGAGGGAG -3' |
| --- | --- |
| Bcl-x Reverse | 5'- ACTGAAGAGTGAGCCCAGCAGA -3' |
| Bcl-xL Forward | 5'-TGAATGAACTCTTCCGGGAT-3' |
| Bcl-xL Reverse | 5'-CCAAGGCTCTAGGTGGTCAT-3' |
| Bcl-xS Forward | 5'-CATATCAGAGCTTTGAACAGGAT-3' |
| Bcl-xS Reverse | 5'-GTCAGGAACCAGCGGTTGAA-3' |
| Actin Forward | 5'-GAGACCTTCAACACCCCAGCC-3' |
| Actin Reverse | 5'-AATGTCACGCACGATTTCCC-3' |

**Supplemental Figure legend**


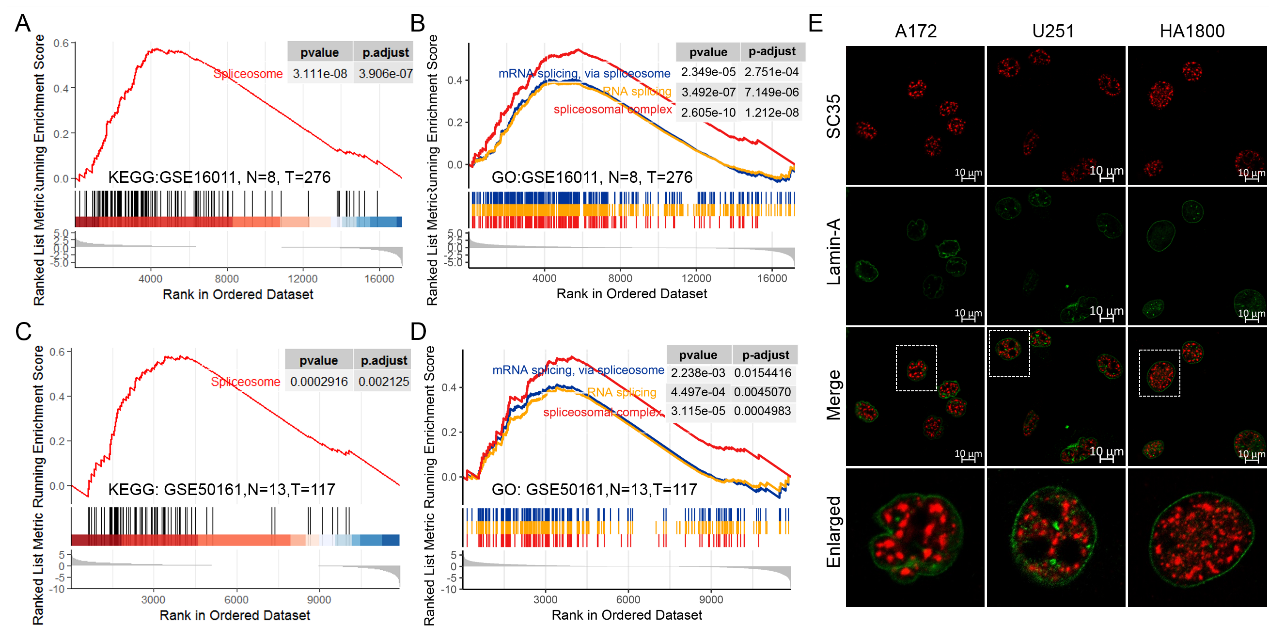


**Supplemental Figure 1.** The disrupted alternative splicing of GBM. (A-D) Gene set enrichment analysis (GSEA) comparing GBM and normal brain (GSE16011, GSE50161) for spliceosome complex and mRNA splicing pathways. (E) Morphologic change of nuclear speckles in GBM (A172, U251) and HA1800 cells. Nuclear speckles were stained with anti-SC35 antibody (red); Anti-lamin A antibody (green) was used to stain nuclear membrane.


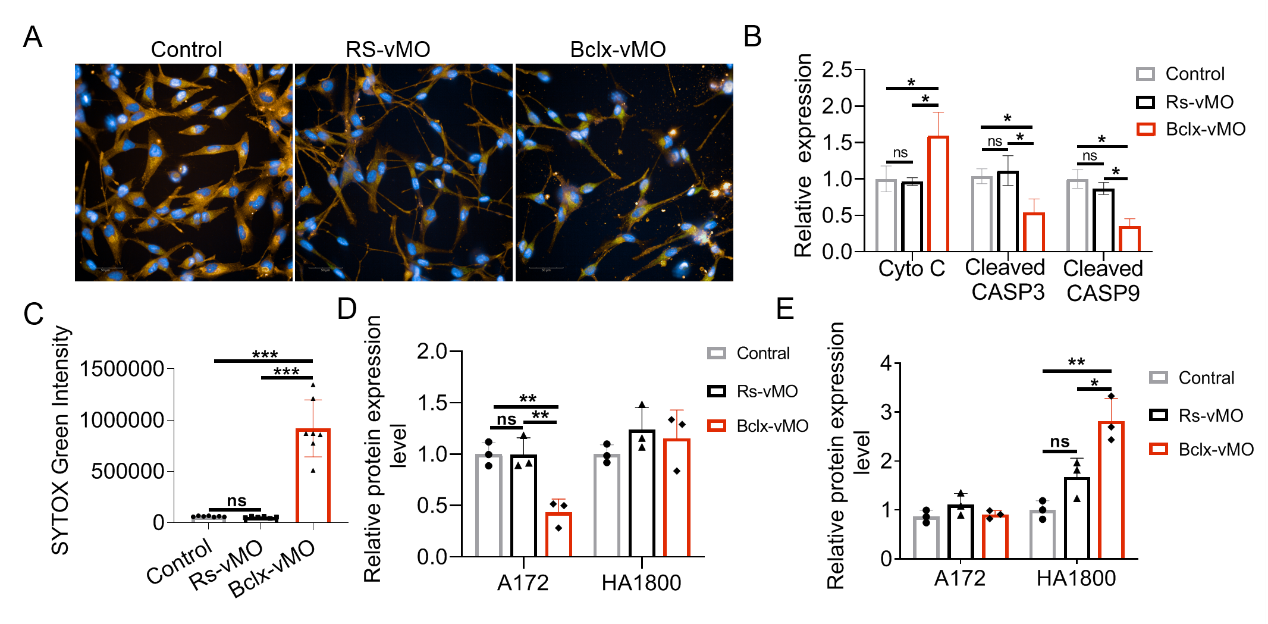


**Supplemental Figure 2.** (A) Fluorescence image of A172 cells stained using JC-10 was ascertained through high content analysis system. (B) Statistical analysis of activated apoptin expression in A172 cell lines treated with vMO for 48h. (C) Statistical analysis of 3D Cell viability of A172 treated with vMO measured using SYTOX Green. (D-E) Statistical analysis of antiapoptotic BCL-2 family protein (BCL-2, MCL1) expression in A172 cell lines treated with vMO for 48h. The concentration of Rs-vMO and Bclx-vMO used was 4μM when not specified. Data are shown as mean values ± S.D. from three independent experiments. One Way ANOVA followed by Dunnett’s multiple comparisons test are reported. For all panels “*” indicates *p*< 0.05, “**” indicates *p* < 0.01, “***” indicates *p* < 0.001, “ns” indicates *p*>0.05.

**
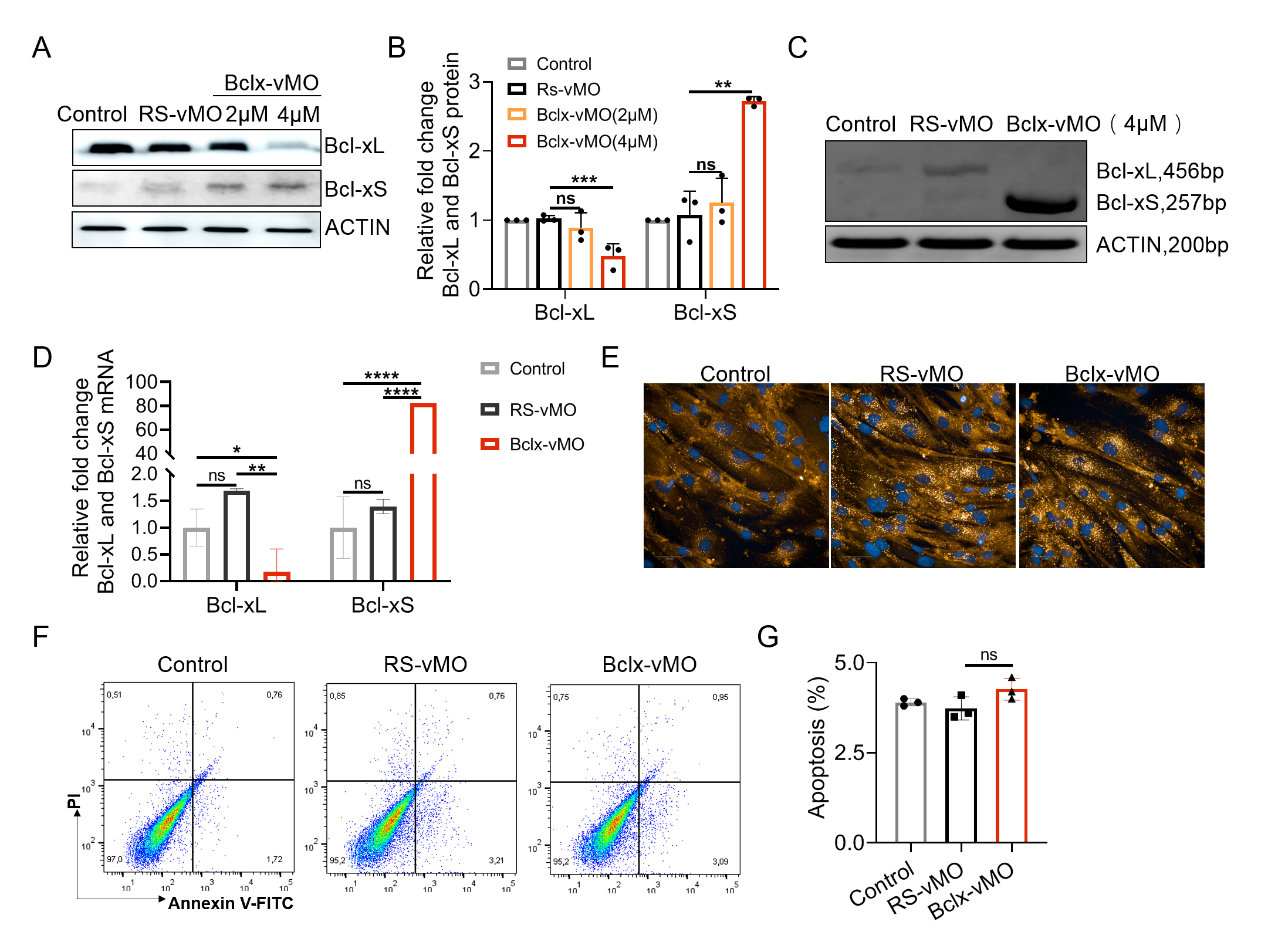
**

**Supplemental Figure 3. *BCLX* splicing regulation by vMO showed no cytotoxicity on normal astrocyte cell HA1800.** (A-B) HA1800 cells were transfected with vMO. The protein expression of Bcl-xL and Bcl-xS were analyzed by western blot. (C-D) HA1800 cells were transfected with vMO. The expression of Bcl-xL and Bcl-xS were analyzed by RT-PCR. (E) Fluorescence image of HA1800 cells stained using JC-10 was ascertained through high content analysis system. (F-G) Apoptosis rate of HA1800 cells received 4uM doses of vMO were detected by flow cytometer. The concentration of Rs-vMO and Bclx-vMO used was 4μM when not specified. Data are shown as mean values ± S.D. from three independent experiments. One Way ANOVA followed by Dunnett’s multiple comparisons test are reported. For all panels “ns” indicates no significance, “*” indicates *p* < 0.05, “**” indicates *p* < 0.01, “***” indicates *p* < 0.001, “****” indicates *p* < 0.0001.


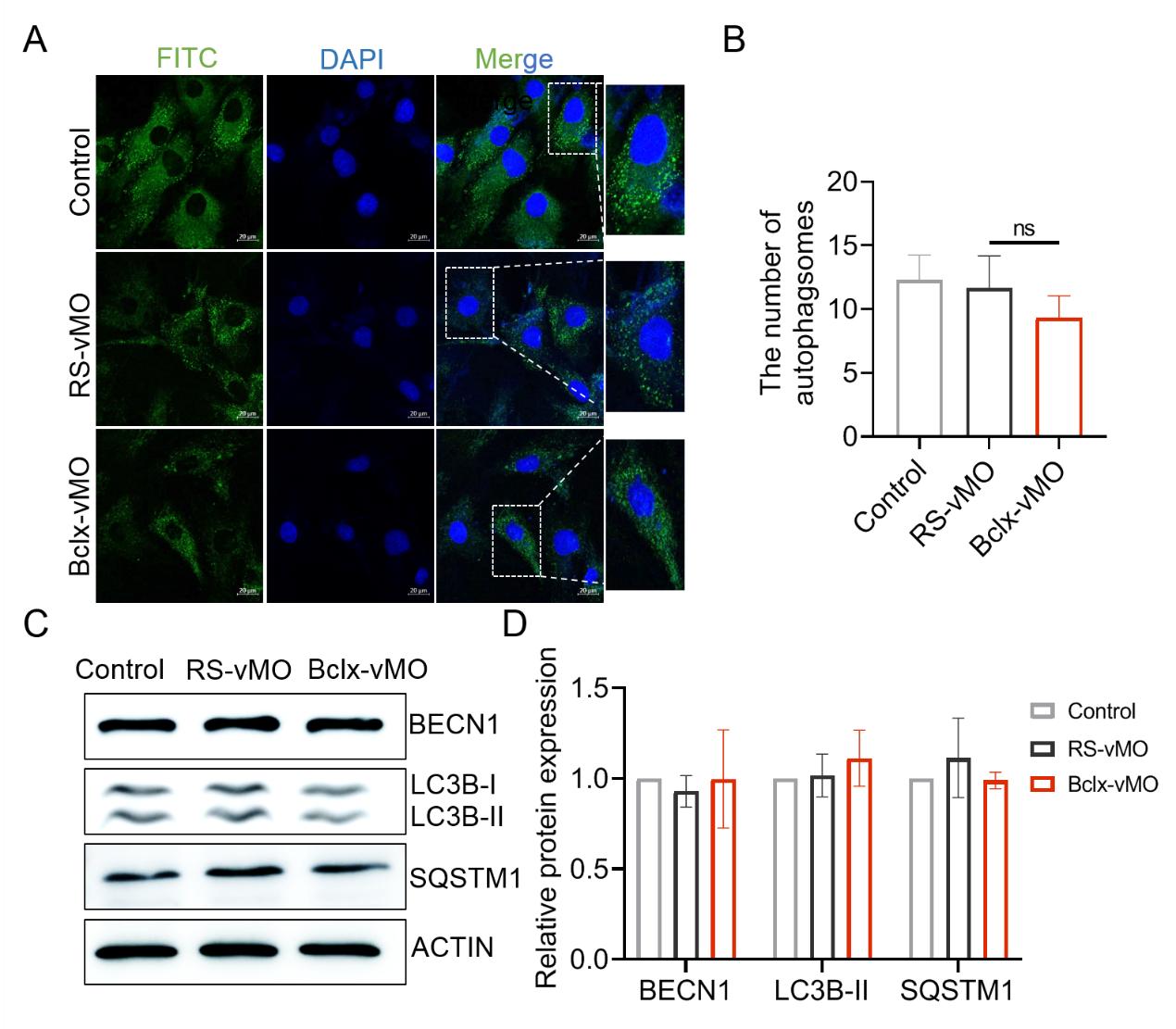


**Supplemental Figure 4. Correction of *BCLX* pre-mRNA alternative splicing induces no autophagy in normal astrocyte cells HA1800.** (A-B) The autophagosomes of HA1800 cells treated with Bclx-vMO were visualized and quantified. (C-D) Expression of BECN1, SQSTM1/p62, and conversion of LC3-I to LC3-II were detected and analyzed through western blotting. The concentration of Rs-vMO and Bclx-vMO used was 4μM when not specified. Data are shown as mean values ± S.D. from three independent experiments. One Way ANOVA followed by Dunnett’s multiple comparisons test are reported. For all panels “ns” indicates *p*> 0.05.


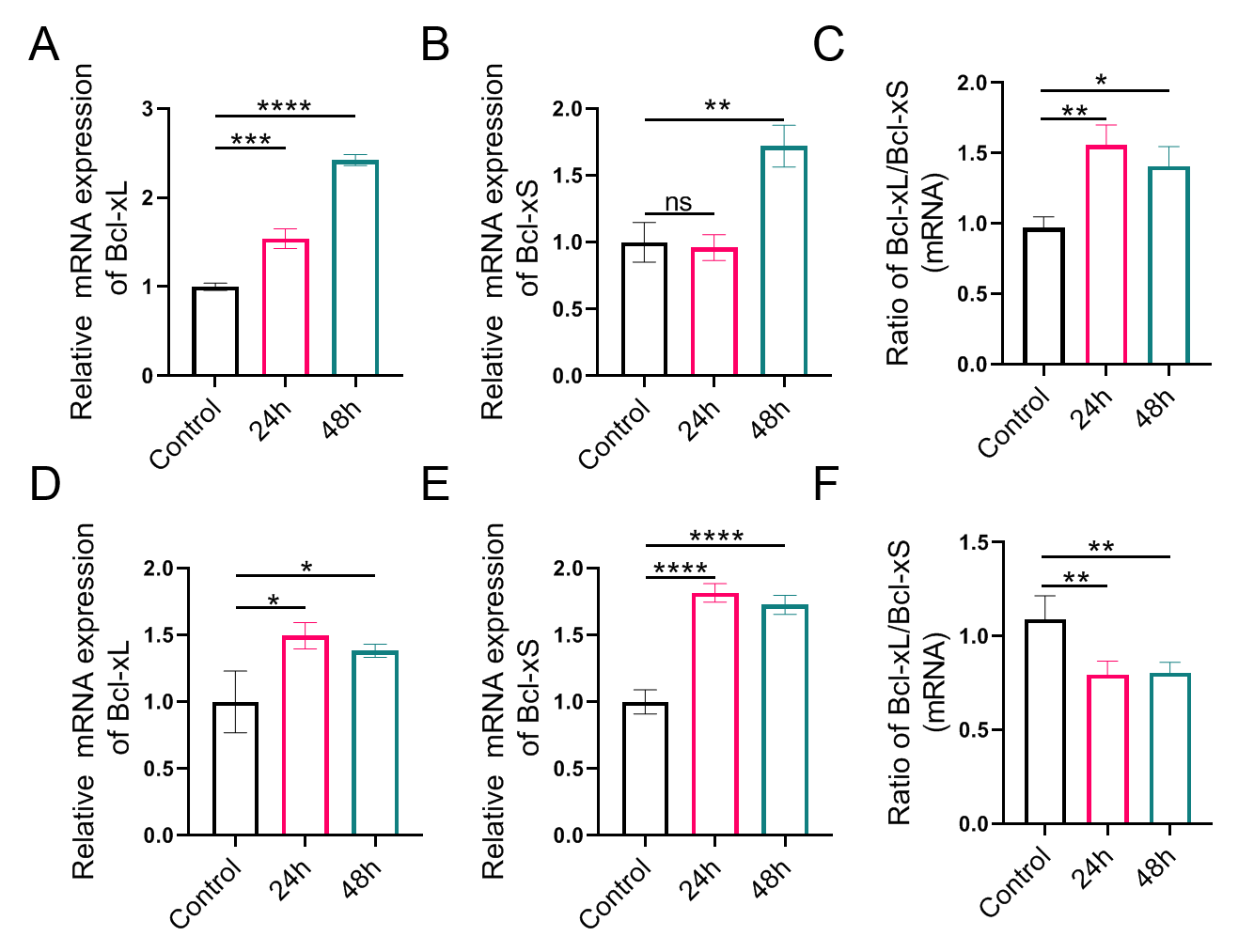


**Supplemental Figure 5. The mRNA expression of Bcl-xL and Bcl-xS after X-ray and carbon ion irradiation by qPCR.** (A-C) The relative mRNA expression of Bcl-xL and Bcl-xS and ratio of Bcl-xL/Bcl-xS after X-ray irradiation. (D-E) The relative mRNA expression of Bcl-xL and Bcl-xS and ratio of Bcl-xL/Bcl-xS after Carbon ion irradiation. Data are shown as mean values ± S.D. from three independent experiments. One Way ANOVA followed by Dunnett’s multiple comparisons test are reported. For all panels, “*” indicates *p*< 0.05, “**” indicates *p*< 0.01, “***” indicates *p*< 0.001, “****” indicates *p* < 0.0001, “ns” indicates *p*> 0.05.


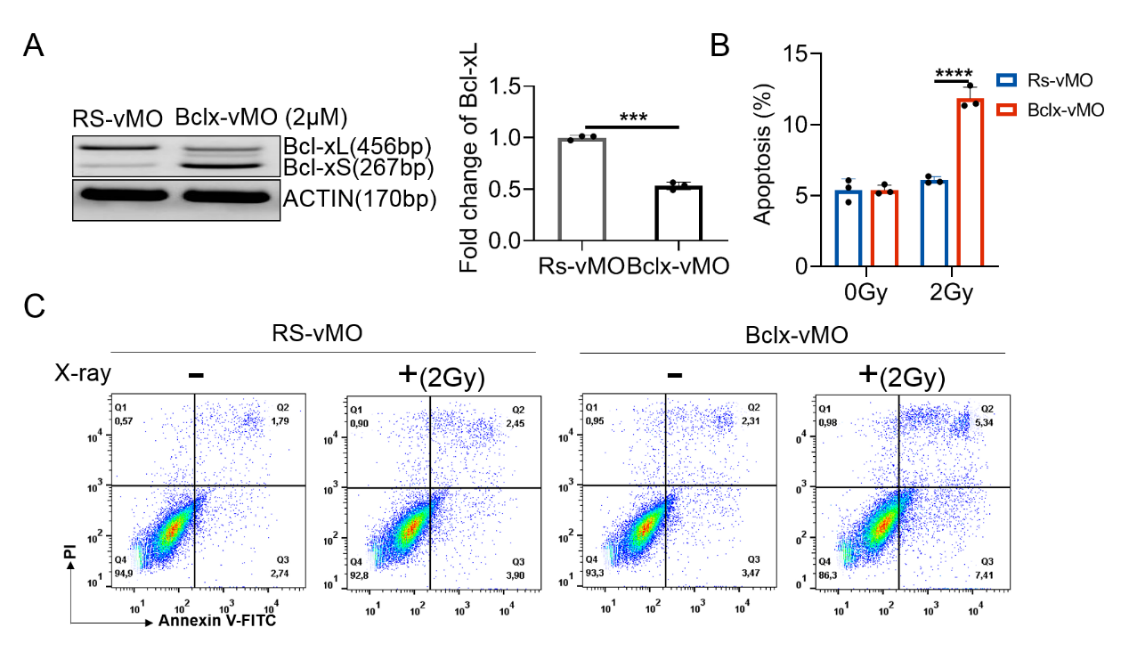


**Supplemental Figure 6. Correction of *BCLX* splicing from anti-apoptotic Bcl-xL to pro-apoptotic Bcl-xS sensitizes GBM cells to X-ray radiation.** (A) The vMO concentration at which the splicing correction efficiency of *BCLX* gene reaches 50% (2μM) was used for radiation sensitization studies. (B-C) Apoptosis of A172 cells with *BCLX* splicing correction or control combined with or without 2Gy X-ray irradiation were evaluated by flow cytometry. The vMO concentration of 2μM was used for radiation sensitization studies. Data are shown as mean values ± S.D. from three independent experiments. One Way ANOVA followed by Dunnett’s multiple comparisons test are reported. For all panels “***” indicates *p*< 0.001, “****” indicates *p* < 0.0001.


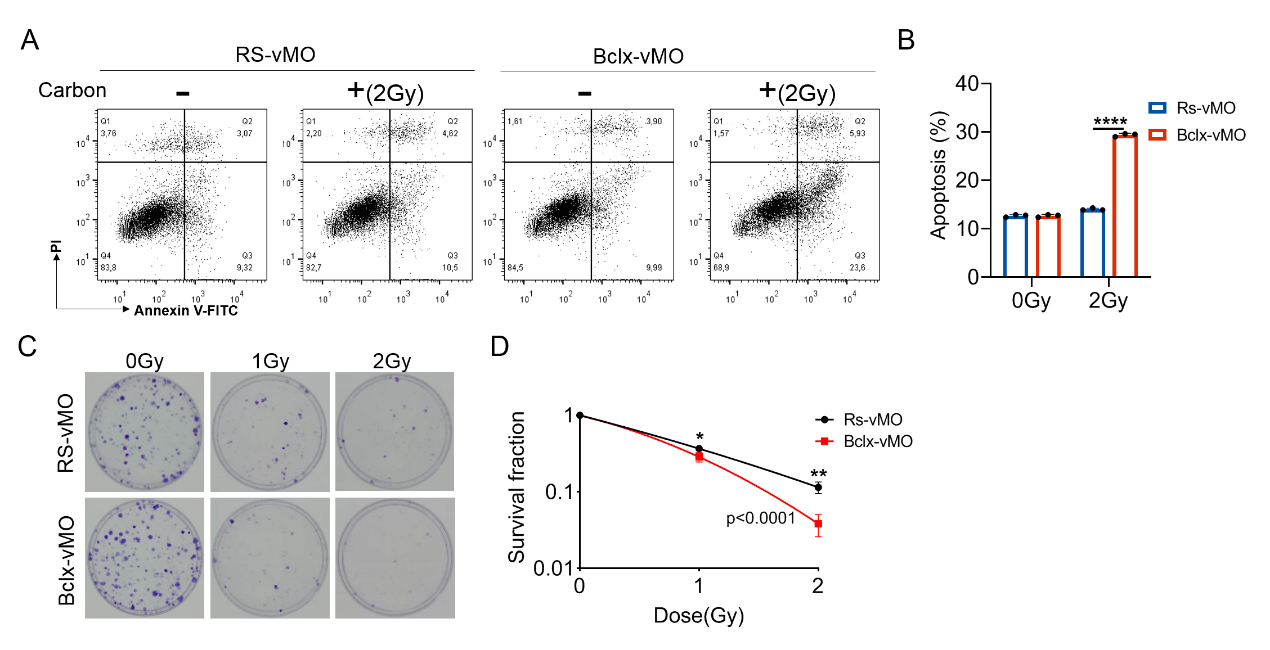


**Supplemental Figure 7. Correction of *BCLX* splicing from anti-apoptotic Bcl-xL to pro-apoptotic Bcl-xS sensitizes GBM cells to carbon ion radiation.** (A-B) Apoptosis of A172 cells with *BCLX* splicing correction or control combined with or without 2Gy carbon ion irradiation were evaluated by flow cytometry. (C-D) Colony formation assays using A172 cells with inhibition of Bcl-xL or control combined with or without carbon ion irradiation. The vMO concentration of 2μM was used for radiation sensitization studies. Data are shown as mean values ± S.D. from three independent experiments. One Way ANOVA followed by Dunnett’s multiple comparisons test are reported. For all panels “*” indicates *p*< 0.05, “**” indicates *p*< 0.01, “****” indicates *p* < 0.0001.


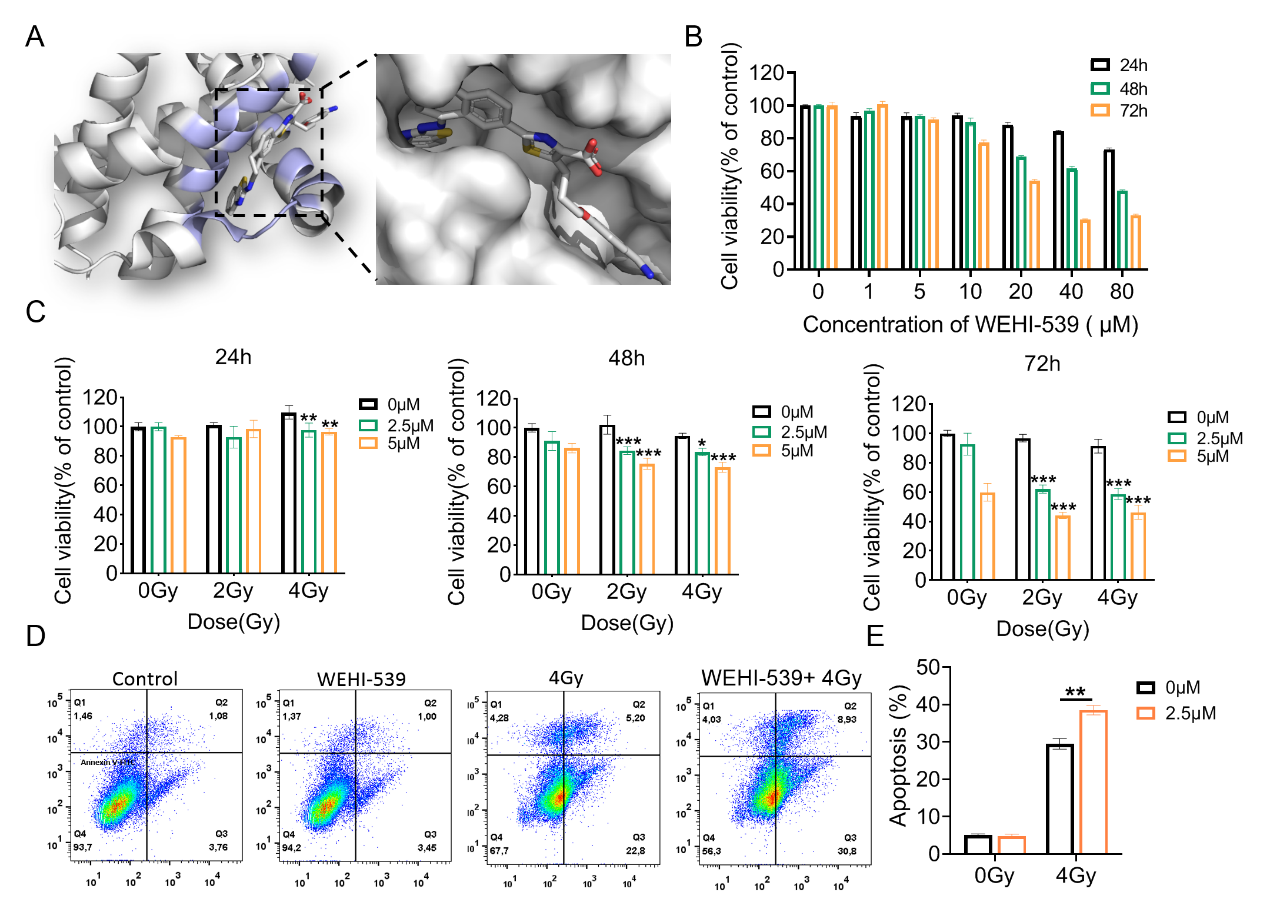


**Supplemental Figure 8. Selective BH3 mimetics WEHI-539 significantly enhanced the radiation sensitivity of A172 cells by inhibition of Bcl-xL.** (A) The structure of WEHI-539 combined with Bcl-xL Hydrophobic groove. (B) The cell viability of A172 cells treatment with concentrations of WEHI-539. (C-D) The cell viability of A172 cells combined treatment of WEHI-539 and X-ray irradiation. (D-E) Apoptosis of A172 cells combined treatment of 0.25μM WEHI-539 and 4Gy x-ray irradiation were evaluated by flow cytometry. Data are shown as mean values ± S.D. from three independent experiments. One Way ANOVA followed by Dunnett’s multiple comparisons test are reported. For all panels “*” indicates *p*< 0.05, “**” indicates *p*< 0.01, “***” indicates *p* < 0.001.
