# Supplementary material for: Modification of BCLX pre-mRNA splicing has antitumor efficacy alone or in combination with radiotherapy in human glioblastoma cells
Source: Cell Death Dis. 2024 Feb 21;15(2):160. doi: 10.1038/s41419-024-06507-x (PMC10881996; doi:10.1038/s41419-024-06507-x)

Figure 1. Anti-apoptotic Bcl-xL is highly expressed in GBM samples and GBM cell lines, and correlated with worse prognosis.

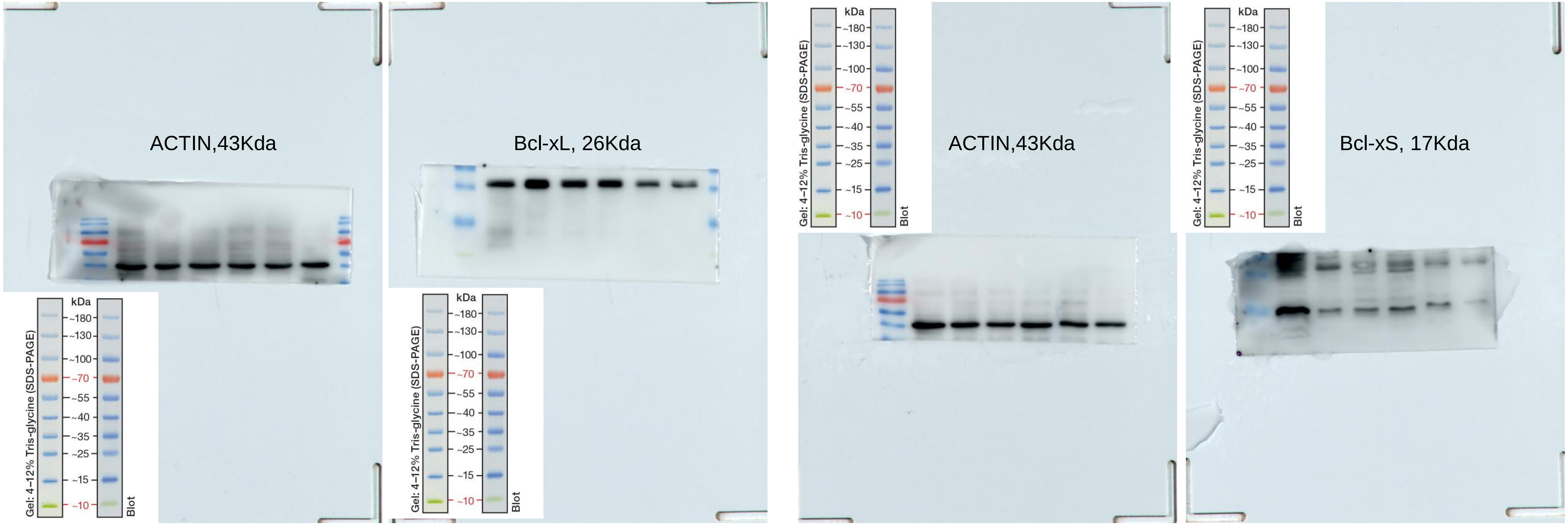

Figure 2. Effect of X-ray and heavy ion irradiation on the alternative splicing and expression of Bclx gene.

Figure. 2A

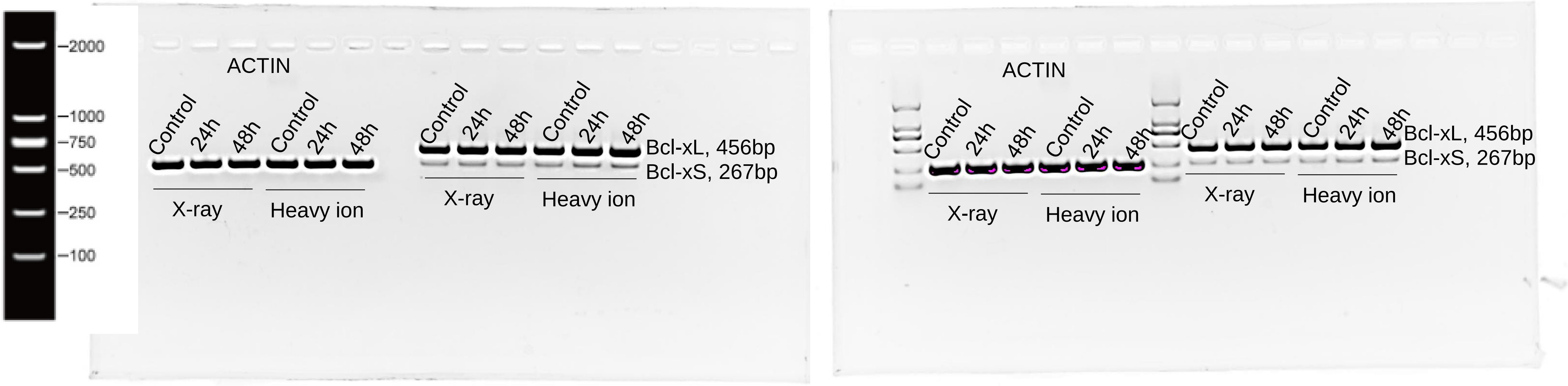

Fig. 2C

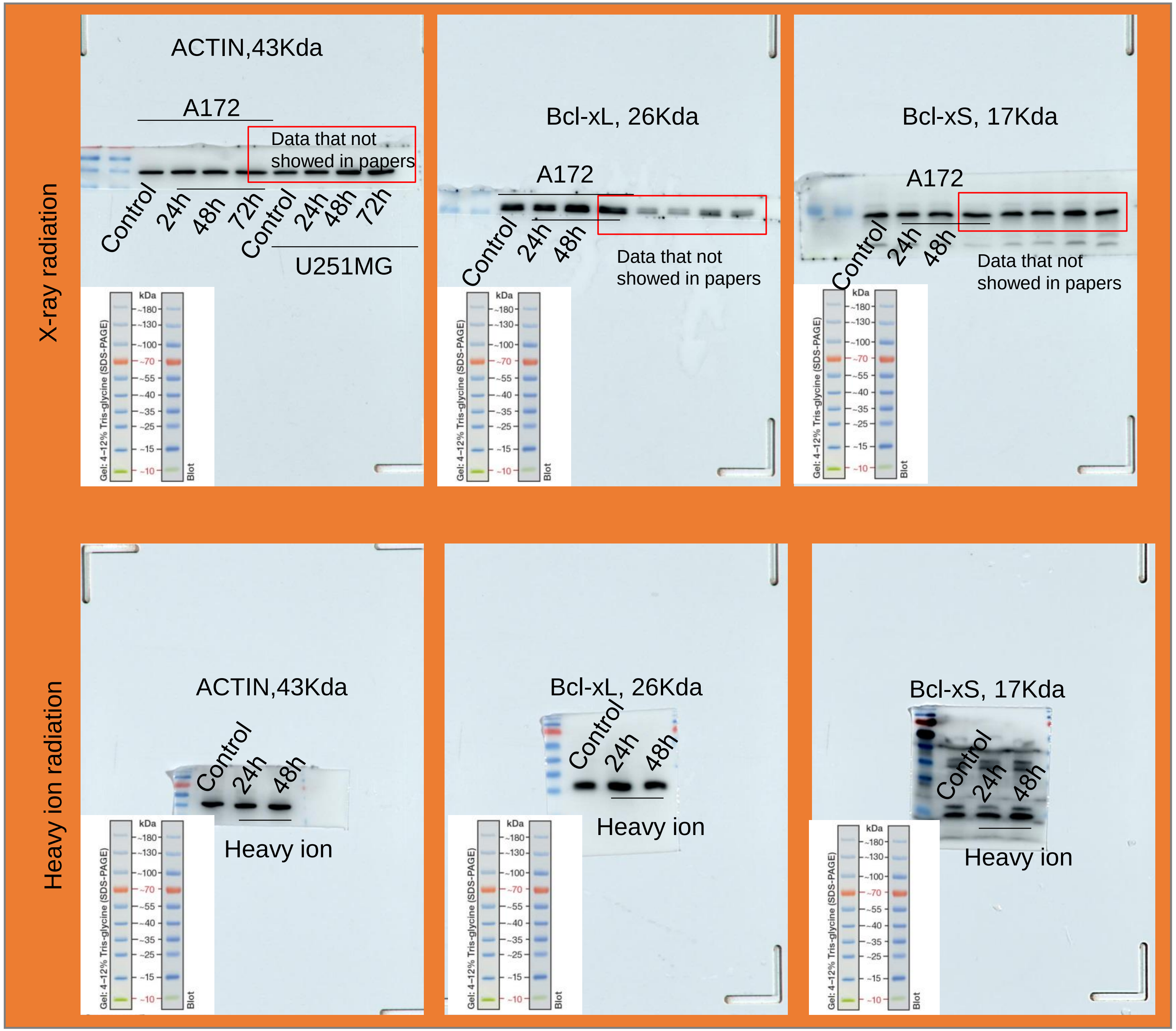

Fig.2G

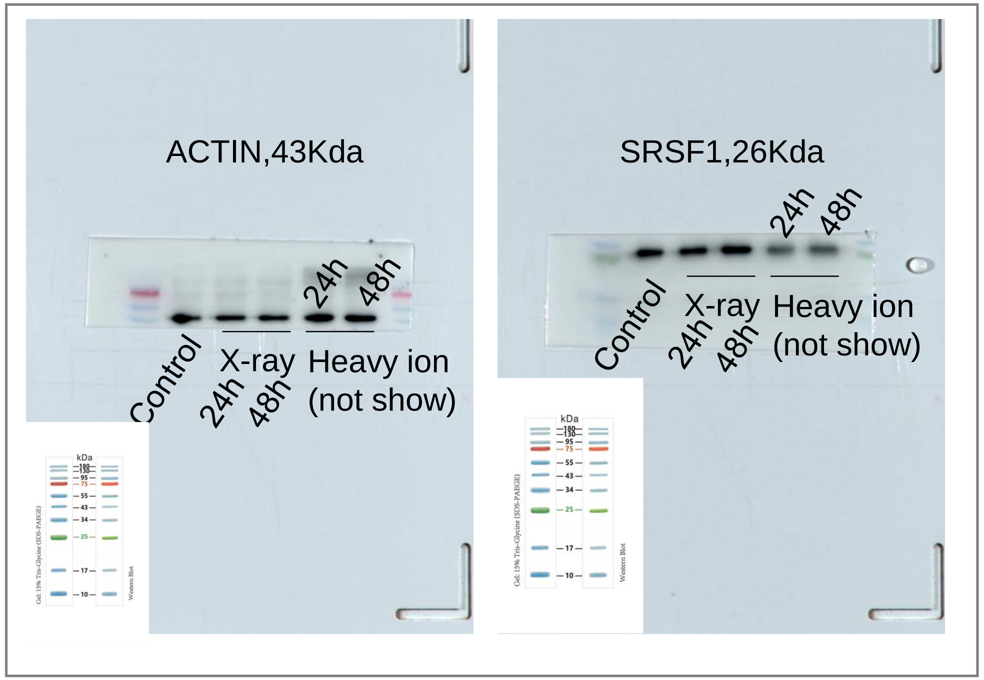

Figure 2. Effect of X-ray and heavy ion irradiation on the alternative splicing and expression of Bclx gene.

Figure 3. vMO regulated Bcl-x splicing mode in GBM cells effectively.

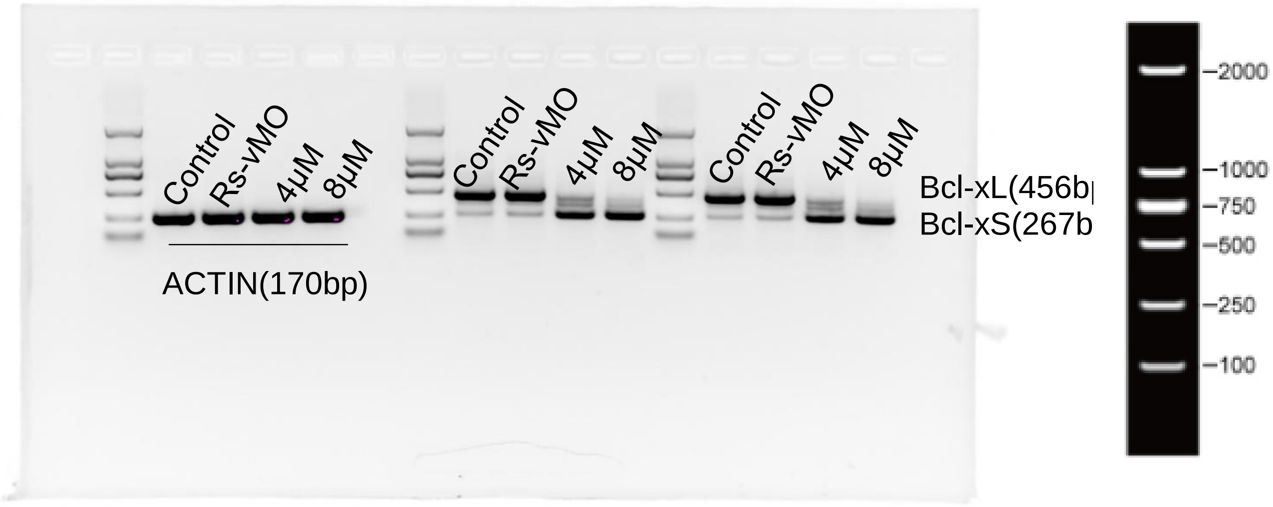

Figure. 3C

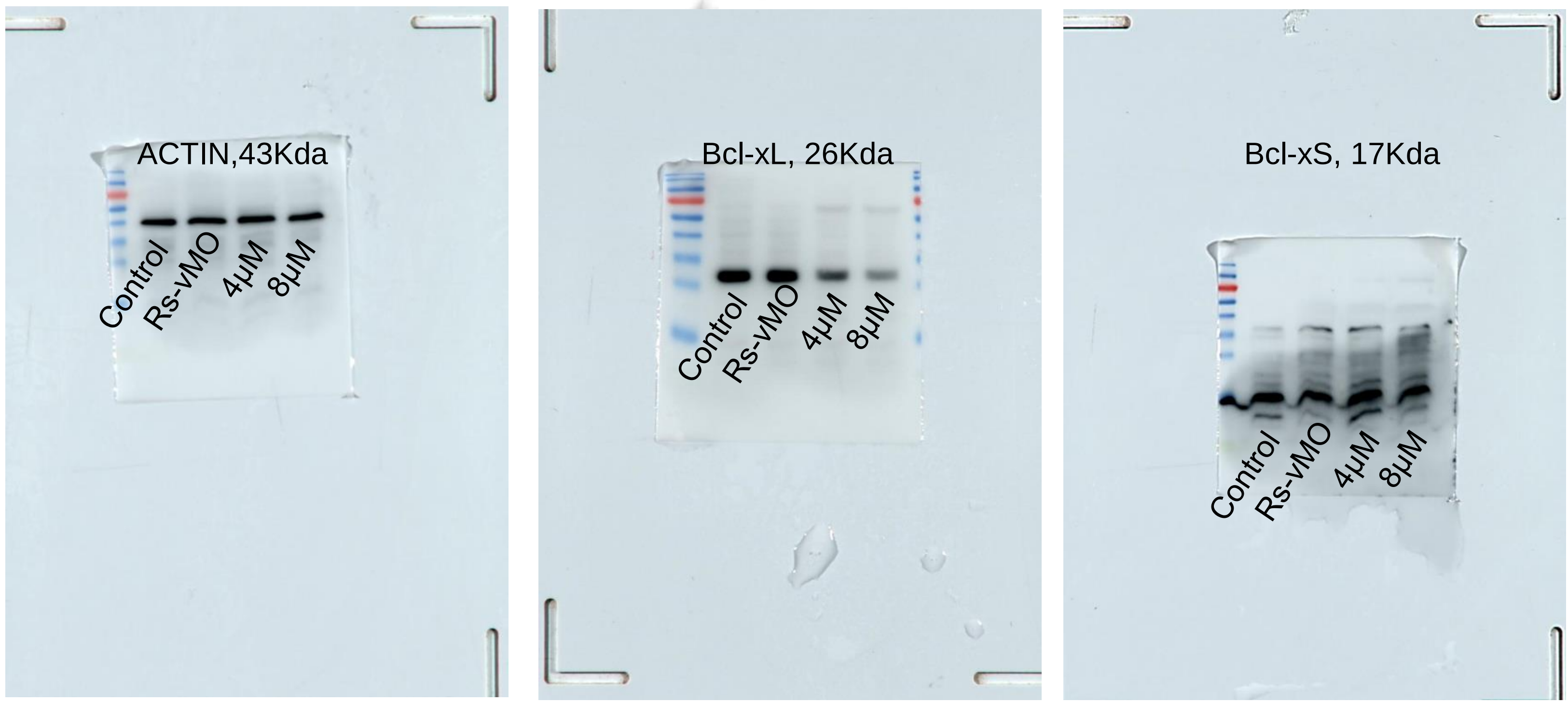

Figure. 3E

Figure 4. Cytotoxicity of GBM cell lines after shift of Bclx Pre-mRNA alternative splicing from Bcl-xL to Bcl-xS.

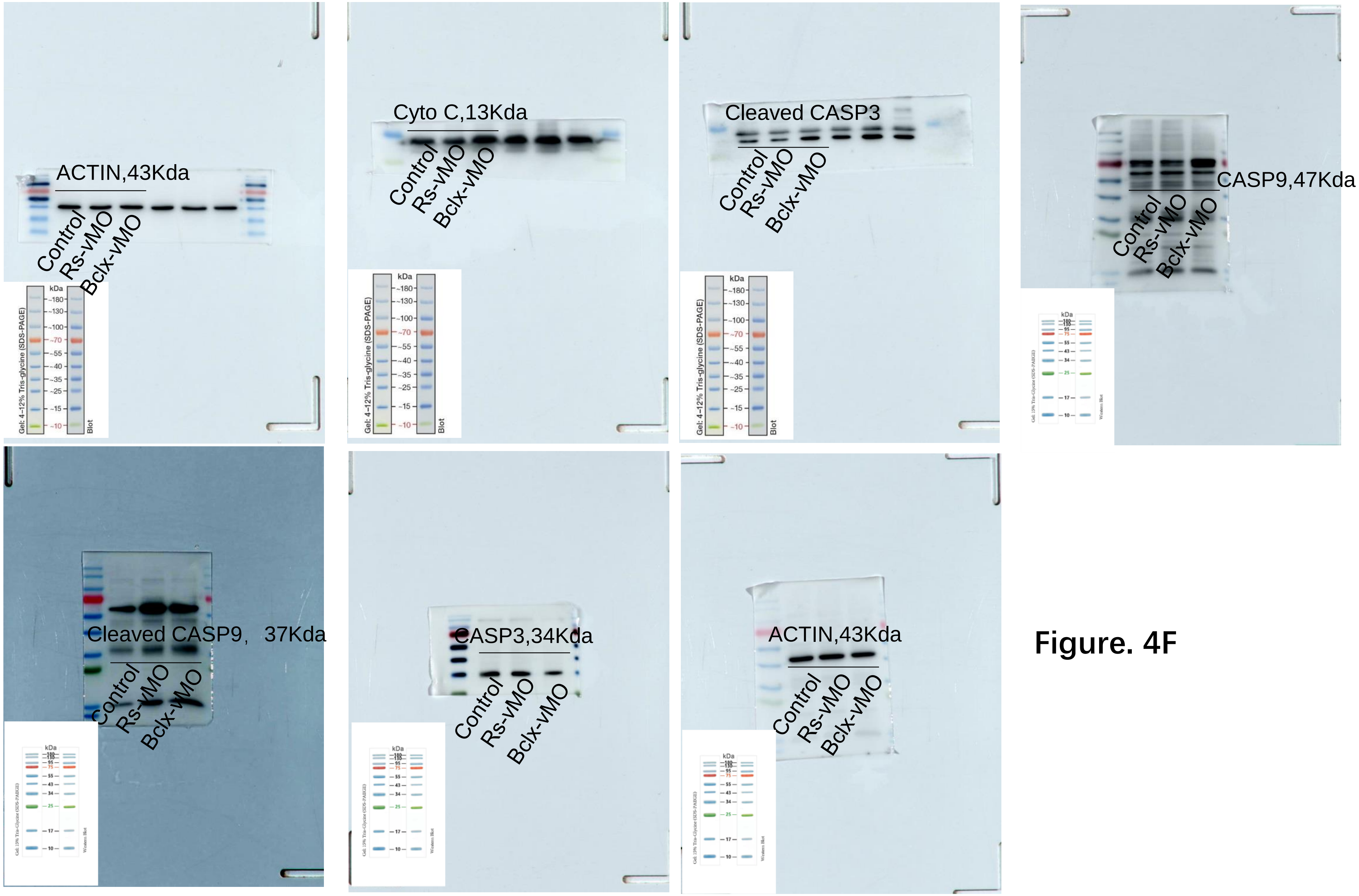

Figure. 4F

Figure. 4I

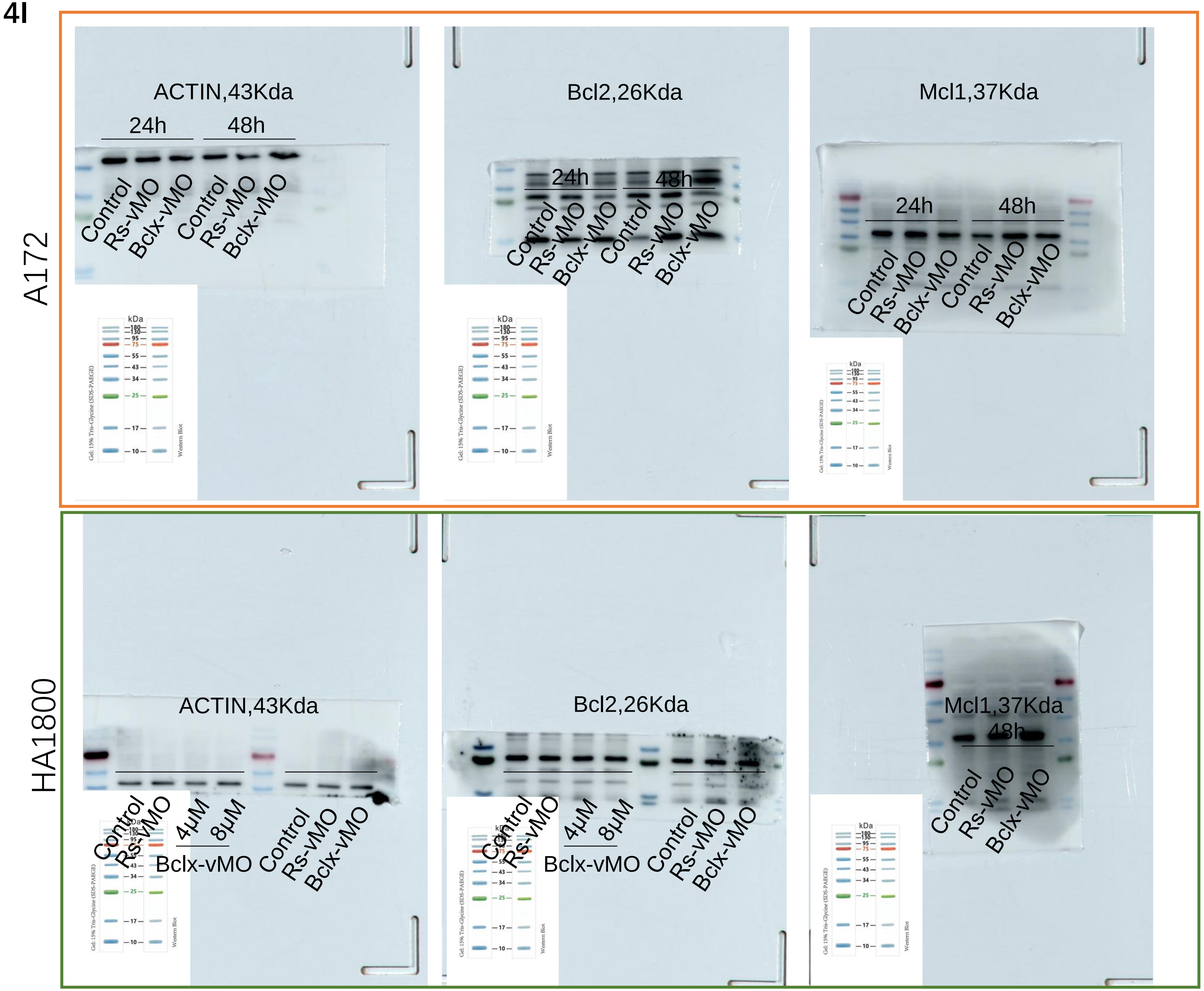
A172
ACTIN,43Kda
24h
48h
Control
Rs-vMO
Bclx-vMO
Control
Rs-vMO
Bclx-vMO
Bcl2,26Kda
24h
48h
Control
Rs-vMO
Bclx-vMO
Control
Rs-vMO
Bclx-vMO
Mcl1,37Kda
24h
48h
Control
Rs-vMO
Bclx-vMO
Control
Rs-vMO
Bclx-vMO
HA1800
ACTIN,43Kda
Control
Rs-vMO
4μM
8μM
Bclx-vMO
Control
Rs-vMO
Bclx-vMO
Bcl2,26Kda
Control
Rs-vMO
4μM
8μM
Bclx-vMO
Control
Rs-vMO
Bclx-vMO
Mcl1,37Kda
48h
Control
Rs-vMO
Bclx-vMO

Figure 5 Correction of Bclx pre-mRNA alternative splicing from Bcl-xL to Bcl-xS induces autophagy in A172 cancer cells.

Figure. 5D

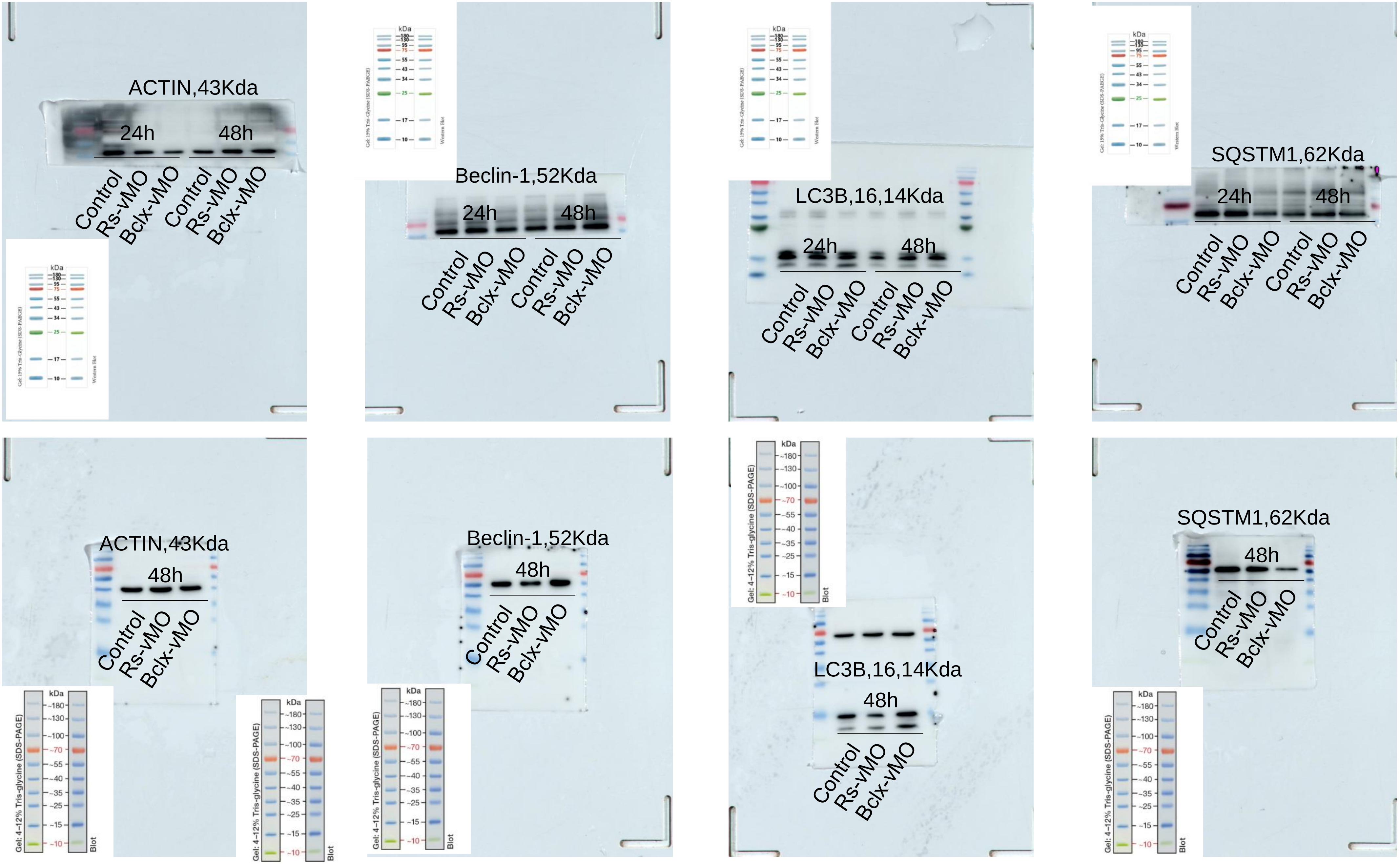

Figure 6 Blocking Bclx splicing regulation-induced autophagy significantly attenuates apoptosis in A172 cells.
Figure. 6E

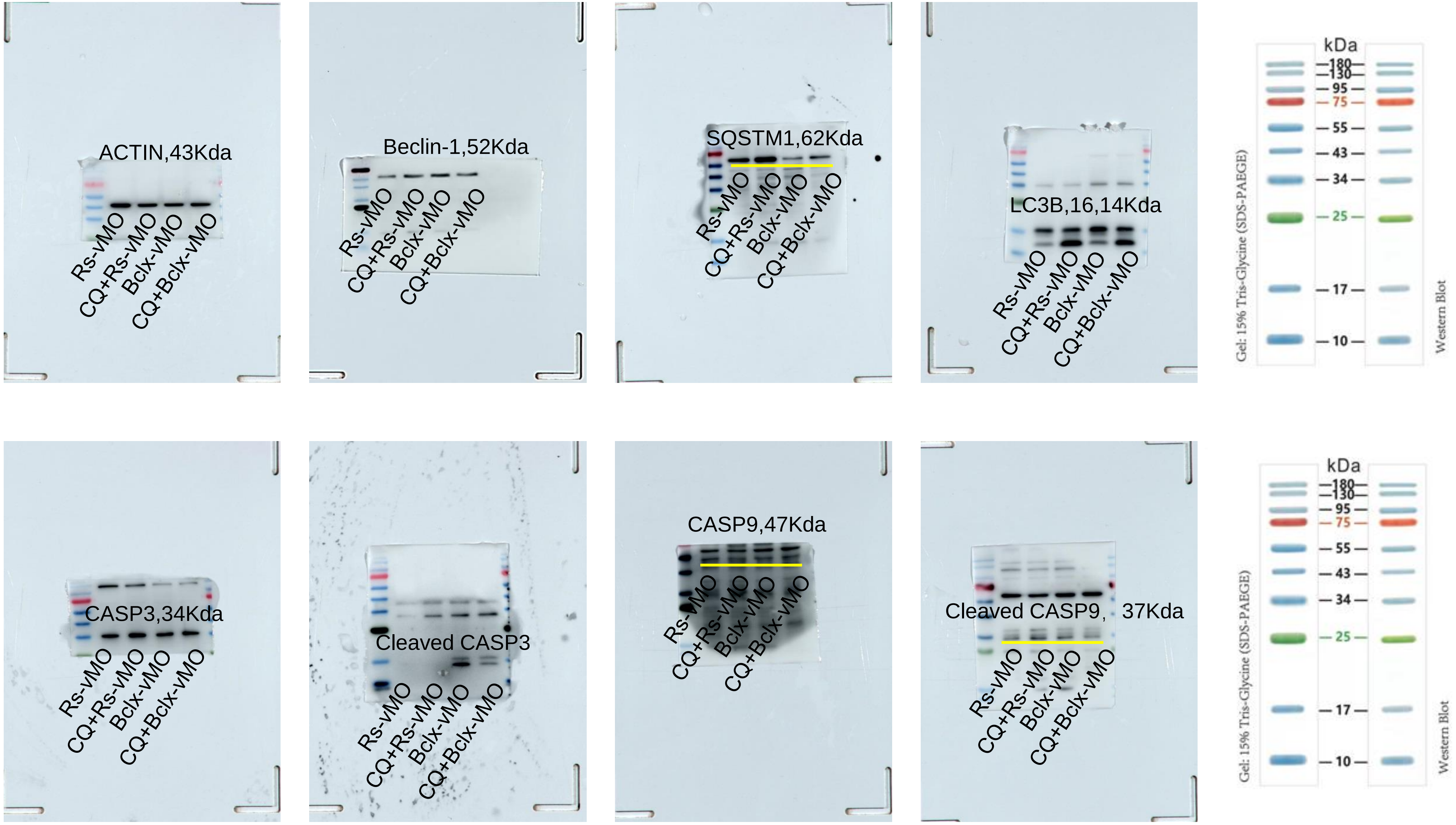

Figure 7 Correction of Bclx splicing from anti-apoptotic Bcl-xL to Bcl-xS sensitizes GBM cancer cells to IR.

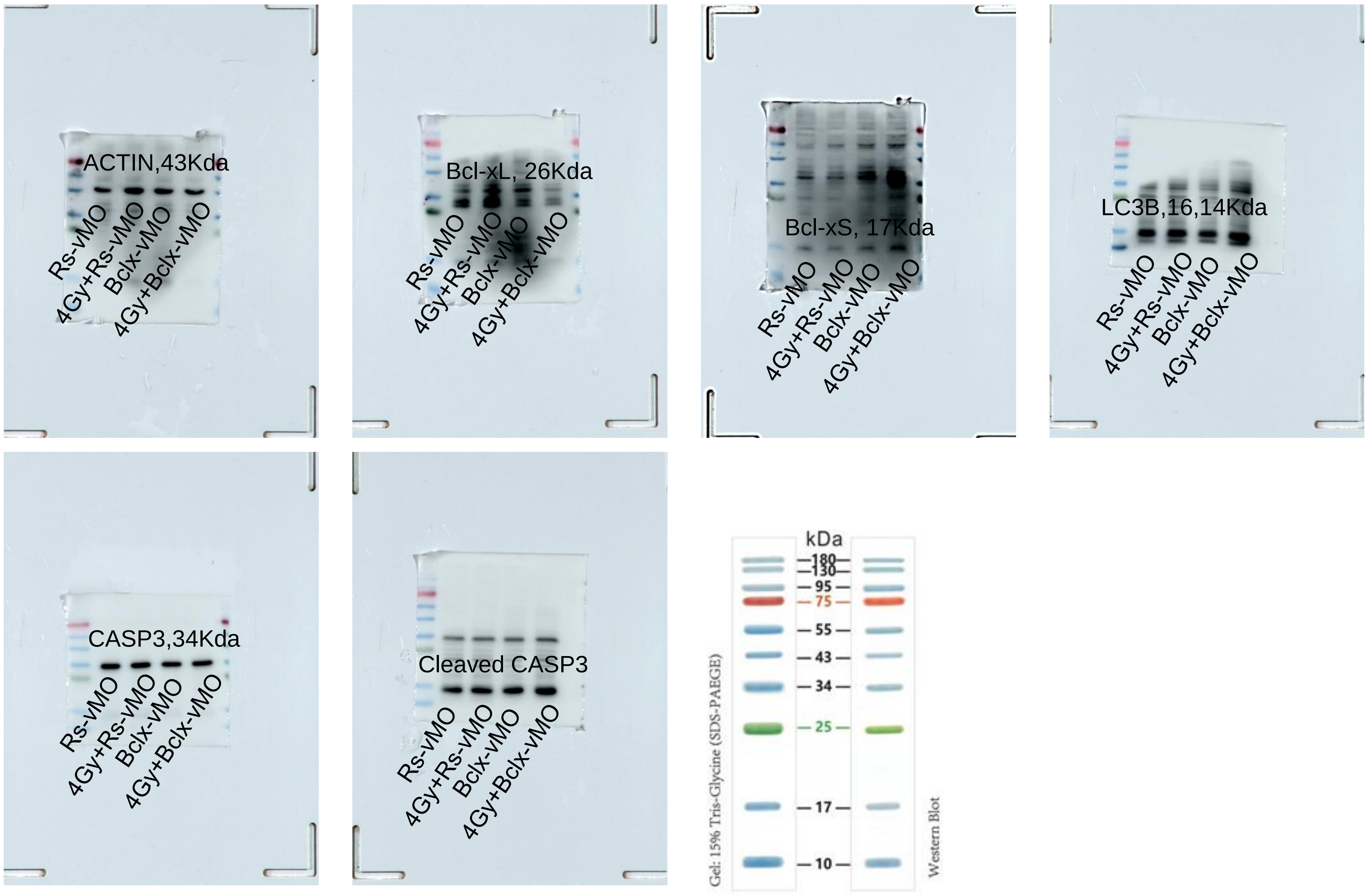

Supplement: Supplementary file 2 — Original Data File [file 41419_2024_6507_MOESM2_ESM.pdf]
